# Soil type and moisture content alter soil microbial responses to manure from cattle administered antibiotics

**DOI:** 10.1007/s11356-024-32903-z

**Published:** 2024-03-20

**Authors:** Sarah Shawver, Satoshi Ishii, Michael S. Strickland, Brian Badgley

**Affiliations:** 1 School of Plant and Environmental Sciences, Virginia Tech, Blacksburg, VA 24061 USA; 2 BioTechnology Institute, University of Minnesota, St. Paul, MN 55108 USA; 3 Department of Soil, Water, and Climate, University of Minnesota, St. Paul, MN 55108 USA; 4 Department of Soil and Water Systems, University of Idaho, Moscow, ID 83844 USA

**Keywords:** Antibiotic resistance, Antibiotic Resistance Genes (ARGs), Soil type, Soil moisture, Manure, Agriculture, Microbial communities

## Abstract

Growing concerns about the global antimicrobial resistance crisis require a better understanding of how antibiotic resistance persists in soil and how antibiotic exposure impacts soil microbial communities. In agroecosystems, these responses are complex because environmental factors may influence how soil microbial communities respond to manure and antibiotic exposure. The study aimed to determine how soil type and moisture alter responses of microbial communities to additions of manure from cattle treated with antibiotics. Soil microcosms were constructed using two soil types at 15, 30, or 45% moisture. Microcosms received biweekly additions of manure from cattle given cephapirin or pirlimycin, antibiotic-free manure, or no manure. While soil type and moisture had the largest effects on microbiome structure, impacts of manure treatments on community structure and individual ARG abundances were observed across varying soil conditions. Activity was also affected, as respiration increased in the cephapirin treatment but decreased with pirlimycin. Manure from cattle antibiotics also increased NH_4_^+^ and decreased NO_3_^−^ availability in some scenarios, but the effects were heavily influenced by soil type and moisture. Overall, this work demonstrates that environmental conditions can alter how manure from cattle administered antibiotics impact the soil microbiome. A nuanced approach that considers environmental variability may benefit the long-term management of antibiotic resistance in soil systems.

**Supplementary Information:**

The online version contains supplementary material available at 10.1007/s11356-024-32903-z.

## Introduction

Antimicrobial resistance (AMR) is a growing concern for both human and animal health (Nesme and Simonet 2015; Sanderson et al. 2016). Antibiotic compounds, ARB, and ARGs are naturally found in soils, and soil ecosystems can serve as a reservoir and possible vector for transmission of AMR (Nesme and Simonet 2015; Fletcher 2015). Application of manure from livestock, whether or not the animal has been administered antibiotics, can result in increased resistance genes and resistant organisms in soils (Udikovic-Kolic et al. 2014; Tasho and Cho 2016). Impacts on ARG abundances in the absence of previous antibiotic exposure are likely due to increases in resistance among native soil organisms caused by nutrients from the manure additions (Udikovic-Kolic et al. (2014). Long-term additions of manure can lead to an increase of ARGs in soil (Fang et al. 2015; Graham et al. 2016; Wepking et al. 2017). ARG abundance generally decreases over time after manure and antibiotic additions (Zhang et al. 2017), but the persistence of antibiotics, and thus selective pressure for ARGs, in soil depends on environmental factors including soil texture, light, temperature, and pH (Tasho and Cho 2016).

In addition to concern about soil acting as a reservoir of AMR persistence, antibiotics can also alter soil microbial communities. Antibiotic additions can decrease microbial abundance and the ratio of bacteria to fungi (Reichel et al. 2014; Lin et al. 2017; Grenni et al. 2018) and reduce overall microbial diversity (Lin et al. 2017; Grenni et al. 2018). Differential effects on survival and abundance of microbial taxa following exposure to antibiotics can also alter the structure of the soil microbiome (Wepking et al. 2017, 2019; Shawver et al. 2021). However, the length and severity of the change can be dependent on the type of antibiotic (Lin et al. 2016). So, while additions of manure from cattle treated with antibiotics can have a variety of effects on soil microbiota, the interacting dynamics among manure addition, antibiotic usage, and environmental change are still poorly understood.

When considering soil as a reservoir for AMR, soils must be considered as heterogeneous ecosystems with many other varying physical, chemical, and biological properties that affect the resident microorganisms. For example, soil texture can influence microbial biomass and community structure (Dequiedt et al. 2009; Roberts et al. 2011; Chau et al. 2011) and even how microbial communities recover after disturbance (Bach et al. 2010). Similarly, soil microbial community structure and diversity are heavily impacted by soil moisture (Lupatini et al. 2019). Different microbial groups respond to soil moisture content in different ways, as fungi have been shown to be more sensitive to small changes in moisture than bacteria (Kaisermann et al. 2015), with greater fungal abundance at higher moisture, with greater bacterial abundance at lower moisture (Borowik and Wyszkowska 2016). Of course, these factors can also interact. For example, de Vries et al. (2012) demonstrated that texture, precipitation, nutrients, and plants all had interactive impacts on soil fungi:bacteria ratios and microbial community composition, with precipitation having the greatest influence.

Soil nutrients are also major drivers of microbial community change, especially in agricultural settings. Inorganic nitrogen and phosphorous amendments as well as organic carbon from plant residues can alter microbial community structure (Leff et al. 2015; Chávez-Romero et al. 2016). Carbon cycling processes such as microbial respiration are also impacted by soil moisture (Manzoni et al. 2012; Kaisermann et al. 2015) and texture (Dijkstra and Cheng 2007). Likewise, soil properties such as texture, pH, and organic carbon (Šantrůčková et al. 2003; Enwall et al. 2010; Deslippe et al. 2014; Morales et al. 2015) and moisture content (Morugán-Coronado et al. 2019) can impact microbially-driven nitrogen cycling processes. With complex interactions of microbial functional responses with environmental factors, more information is required to understand how these processes are impacted by AMR.

Given the dominant importance of environmental factors in broadly mediating microbial processes, it is critical to better understand how they specifically mediate interactions among antibiotics, resistant organisms, and microbial communities. Soil moisture can play an important role here as well, as sulfonamide resistance gene abundances have been shown to be negatively correlated with temperature and precipitation (Zhou et al. 2017), and fluctuations in soil moisture in combination with sulfadiazine can differentially affect some microbial functional groups (*e.g.,* nitrifiers but not denitrifiers; Radl et al. 2015). Likewise, soil texture can mediate antibiotic impacts on soil communities by affecting the binding and persistence of antibiotic compounds. Soil texture can also influence how long pathogens from manure, which are potentially ARB, survive in soil, with longer survival in clay soils (Sharma and Reynnells 2016). However, evidence of the effect of texture is inconsistent, with some studies suggesting that finer textured soils will bind to antibiotics more strongly and have a greater impact on soil microbial communities (Chander et al. 2005), while others suggest that antibiotics in coarse soils will cause greater shifts in microbial community structure and abundance of ARGs (Pankow 2017; Chen et al. 2017, 2018; Blau et al. 2018). These results may differ because soils usually vary in more than just texture. They may also differ in the mineral composition, pH, organic matter content, and nutrient concentrations, which could all impact results.

A common characteristic of early work studying the influence of antibiotics, manure, and soil properties on soil microbial communities is that only one soil property is studied at a time, such as texture (Chander et al. 2005; Chambers et al. 2015; Sharma and Reynnells 2016; Pankow 2017; Blau et al. 2018; Chen et al. 2018), moisture (Reichel et al. 2014; Radl et al. 2015; Jechalke et al. 2016), or temperature (Lin et al. 2017). Thus, little information exists on the interactive effects of soil properties on soil microbial structure, functional response, and ARG abundances impacted by manure additions. The purpose of this study was to (1) examine the impact of soil moisture and type on ARG abundances and microbial community structure during the addition of manure from cattle treated with antibiotics and (2) identify changes in microbial respiration and inorganic soil nitrogen pools among these same treatments. Overall, we expected soil type and moisture to be primary controls of ARG abundances, microbiome structure, and function, but that manure from cattle with and without previous treatment with antibiotics would have significant impacts within similar environments. We also expected significant interactions would indicate that soil moisture and type would alter microbiome responses to manure from cattle administered antibiotics. Finally, we expected that ARG abundances would respond to the addition of manure alone, even without a history of antibiotic exposure.

## Methods

### Study design

This work is part of a larger project designed to understand the real-world implications of applying manure from cattle treated with antibiotics on soil processes and microbial communities (Wepking et al. 2019; Shawver et al. 2021). The benefit and rationale for this approach is that it mimics the typical exposure pathway connecting agricultural soils to antibiotics used to treat livestock, as opposed to the many studies that have investigated direct application of antibiotic compounds to soil. This approach realistically accounts for the impact administering antibiotics has on the contents of the manure, including excreted antibiotics, metabolites, antibiotic-resistant bacteria and ARGs. However, it is important to acknowledge the inherent tradeoff in this approach, namely that the amount of antibiotics present in manure is not controlled in the current study.

This study was conducted as a microcosm experiment with three factors: type, moisture, and manure. Soil was collected from Virginia Tech’s Eastern Shore Agricultural Research and Extension Center (SL; 37.585602, − 75.823327) and the Reynolds Homestead Forestry Research Center (SCL; 36.644596, − 80.148816). The SL soil was mapped as Bojac sandy loam, and the SCL soil was mapped as Fairview sandy clay loam (Soil Survey Staff (2019). It is important to note that while the soils were originally chosen for their textures, they had multiple differences in soil properties (Table 1; Supplementary Table 1). Therefore, throughout this study, the term “type” is used to distinguish the two soils. Soils were air dried, ground, sieved to 2 mm, and thoroughly homogenized. The gravimetric moisture treatments used in this study were 15, 30, and 45% wt/wt. These values were chosen to cover a range of dry to moist conditions for both soils. The manure treatments were a control manure from cattle that did not receive antibiotics (CO), manure from cattle administered cephapirin benzanthine, a bactericidal cephalosporin (CE), manure from cattle treated with pirlimycin hydrochloride, a bacteriostatic lincosamide (PI), and a no manure control (NM). Five replicate microcosms of each type, moisture, and manure combination were used. Manure was collected as described in Wepking et al. (2019). Briefly, manure was collected from cattle two to three days following administration of antibiotics. The collected manure was homogenized and stored at -20 °C until use due to differences in timing of manure collection from different livestock. Freezing was used to preserve the antibiotic compounds and metabolites and generally prevent the decomposition of the manure as much as possible. However, it is important to note that it likely had some impact on how many viable bacteria were introduced to the soil microcosm. Subsamples of soils and manure additions from before the start of the experiment were frozen at -80º C for analysis.

**Table 1:** Soil data from the 2 soil samples used in the study. Map unit and texture data were obtained from the Soil Survey (Soil Survey Staff 2019), while organic matter (OM), pH, and cation exchange capacity (CEC) were measured from soil samples

Soil	Map unit	Texture	Sand %	Clay %	OM %	pH	CEC (meq 100g^–1^)
SL	Bojac sandy loam	Sandy loam	65	10	0.8	5.8	3.1
SCL	Fairview sandy clay loam	Sandy clay loam	58	26	3.8	6.3	5.2

For each microcosm, 15 g of soil (dry weight) was added to a 50 mL conical centrifuge tube. Autoclaved deionized water was added to each tube to achieve the correct moisture content. Tubes were loosely capped to allow for gas exchange and incubated in the dark at room temperature (~ 22 °C). Moisture content was maintained by replacing water based on mass loss twice per week, accounting for any water added in with manure amendments. Soils were incubated for 5 days at the designated moisture content for each treatment prior to starting the experiment to allow initial adjustment of soil microbial communities. Manure was applied at 10 g/kg soil (wet weight) every other week for 10 weeks for a total of five applications. The application rate was based on inputs from an average density of stocking dairy cattle as used in previous studies (Wepking et al. 2019; Shawver et al. 2021). Soils were mixed thoroughly after manure additions to ensure homogenization. Soils for NM microcosms were also mixed to maintain consistency in microcosm handling.

### Antibiotic-resistance genes

Microfluidic quantitative PCR (mfqPCR) was used to quantify the absolute abundances of a wide variety of genes, including integrase, metal-resistant, and several classes of antibiotic resistance genes, including tetracycline, sulfonamide, β-lactamase, and lincosamide resistance genes, along with metal resistance and integrase genes. A full list of genes can be found in Table S1. Protocols were done following the methods in Ahmed et al. (2018) and Sandberg et al. (2018). In brief, specific target amplification (STA), a 14-cycle multiplex PCR with all primers used for the mfqPCR, was performed prior to mfqPCR to increase the amount of DNA template as described previously (Ahmed et al. 2018). Both DNA samples and the standard plasmid mixture (2 × 10^0^ to 2 × 10^6^ copies/μL) were subjected to the STA reaction. The STA products (8 μL) were diluted five-fold by adding 32 μL of TE buffer (10 mM Tris–HCl and 0.1 mM EDTA [pH = 8]) and used for the mfqPCR. The mfqPCR was done using the BioMark HD Real-Time PCR system (Standard BioTools) with the 96.96 GE Dynamic Array IFC as described previously (Ahmed et al. 2018). The threshold cycle (C_T_) was determined using Real-Time PCR Analysis Software ver. 3.0.2 (Standard BioTools). The standard curves were generated by linear regression analysis of the C_T_ values vs. the amounts of the template DNA (log copies/µL) as described previously (Ishii et al. 2013). Samples were compared to standard curves and normalized to log copies/g soil. Raw sequence reads are archived under BioProject ID PRJNA843096.

### Microbial community structure

Aliquots of frozen soil collected at the end of the 10-week experiment were analyzed for microbial community structure and ARG abundances. Total DNA was extracted from soil samples using Qiagen PowerSoil DNA extraction kits (Qiagen, Hilden, Germany.). The V4 region of the 16S rRNA gene was amplified in triplicate for bacterial community composition, following Earth Microbiome protocols (Caporaso et al. 2012). Bacterial primers were 515F (5’-GGA CTA CNV GGG TWT CTA AT-3’) (Parada et al. 2016) and 806RB (5’-GTG YCA GCM GCC GCG GTA A-3’) (Apprill et al. 2015). Triplicate amplifications for each sample were pooled and visualized on agarose gel with no template controls for each tagged primer to verify no contamination was present. PCR products were purified with QIAquick PCR purification kits, and cleaned amplicons were sequenced on the Illumina MiSeq platform with 300 bp single reads.

Raw sequence reads were processed with QIIME version 2 (Bolyen et al. 2019). Sequences were then processed into Amplicon sequence variants (ASVs) using DADA2 (Callahan et al. 2016). Denoising criteria for DADA2 included trimming the first 13 bp due to low quality and truncation at 250 bp for both forward and reverse reads. Otherwise, default criteria were used, including truncating reads at any base position where the quality score Q < 2 and discarding any sequences with ambiguous bases or expected errors > 2.0. Amplicon sequence variants (ASVs) were then identified with a trained naïve Bayes classifier (Pedregosa et al. 2011) and GreenGenes 13.8 (DeSantis et al. 2006) reference database. ASVs identified as chloroplast or mitochondria DNA were discarded.

### Microbial respiration and soil nitrogen

To investigate treatment impacts on microbial function, soil respiration, active microbial biomass, and changes in soil dissolved nitrogen pools were all quantified. During the experiment, soil respiration was measured weekly. Microcosm tubes were flushed with CO_2_-free air, capped tightly, and incubated at room temperature for 24 h. At the end of the 24-h incubation, accumulated CO_2_ in the headspace was measured using a bench-top infrared gas analyzer (IRGA, LI-7000 CO2 H2O Analyzer, Li-Cor, Lincoln, NE). Soil respiration incubations were conducted immediately following manure applications and at the same time the following week to capture both the responses to manure and antibiotic additions and the background respiration throughout the experiment, respectively. At the end of the 10-week incubation, substrate induced respiration (SIR) was measured as an estimate of active microbial biomass as described in supplementary information (Wepking et al. 2017).

Following the 10-week incubation, aliquots of soil were set aside and stored at − 80 °C for N extractions. Soils and manure from the start of the experiment were also measured to estimate N concentrations at the start of the experiment and quantify how much N was added with manure. Soils were thawed and 2.5 g (dry weight) was placed in 50 mL conical centrifuge tubes along with 25 mL of 2 M KCl and shaken for 1 h. Samples were then centrifuged and the supernatant was decanted and filtered. The filtrate was stored at 4ºC until analyzed (less than 24 h). The filtrate was analyzed for NH_4_^+^ and NO_3_^−^ on a Lachat Instruments QuickChem 8500 auto-analyzer (Lachat Instruments, Hatch Company, Loveland, CO) using QuickChem methods 12–107-06–2-A for NO_3_^−^ and 12–107-04–1-B for NH_4_^+^ (Hofer 2003; Knepel 2003). To account for N added with manure, NO_3_^−^ and NH_4_^+^ results are presented as percent change, calculated by dividing the total mass of NH_4_^+^ or NO_3_^−^ in the samples at the end of the incubation by the mass in the soil at the start of the experiment plus the total mass added with manure.

### Statistical analysis

Differences in respiration rates and NO_3_^−^ or NH_4_^+^ concentrations were tested using 3-way ANOVA with Tukey’s HSD tests for pairwise comparisons. The 16S dataset was analyzed in R (version 3.5.1 (R Core Team 2018)) using *vegan* (Oksanen et al. 2019) and *phyloseq* (McMurdie and Holmes 2013). Differences in microbial community structure among treatments, soil type, and moisture were visualized with principal coordinates analysis (PCoA) of Bray–Curtis distance matrices. Differences in overall microbial community structure were tested using PERMANOVA with the function “adonis” in the package *vegan* (Oksanen et al. 2019). Post-hoc pairwise comparisons were made with “pairwise.adonis” in the package *pairwiseAdonis* (Arbizu 2017). Taxa that were responsible for changes in community structure were determined with indicator species analysis, using “multipatt” in the package *indicspecies* (De Cáceres et al. 2020)*. P* values were adjusted for multiple comparisons using FDR-adjusted *P*-values.

Overall patterns in ARG abundances were also visualized using PCoA and tested using “adonis” and “pairwise.adonis.” To determine which ARG abundances changed among samples, nonparametric factorial ANOVAs using the function “art” in the package *ARTool* (Kay et al. 2021) were used with FDR-adjusted p-values for each gene. For genes that were significantly different for one or more of the main effects (manure treatment, soil type, or moisture content), post hoc analyses were conducted using the function “art.con” in the package ARTool.

## Results

### ARGs

**Fig. 1:**
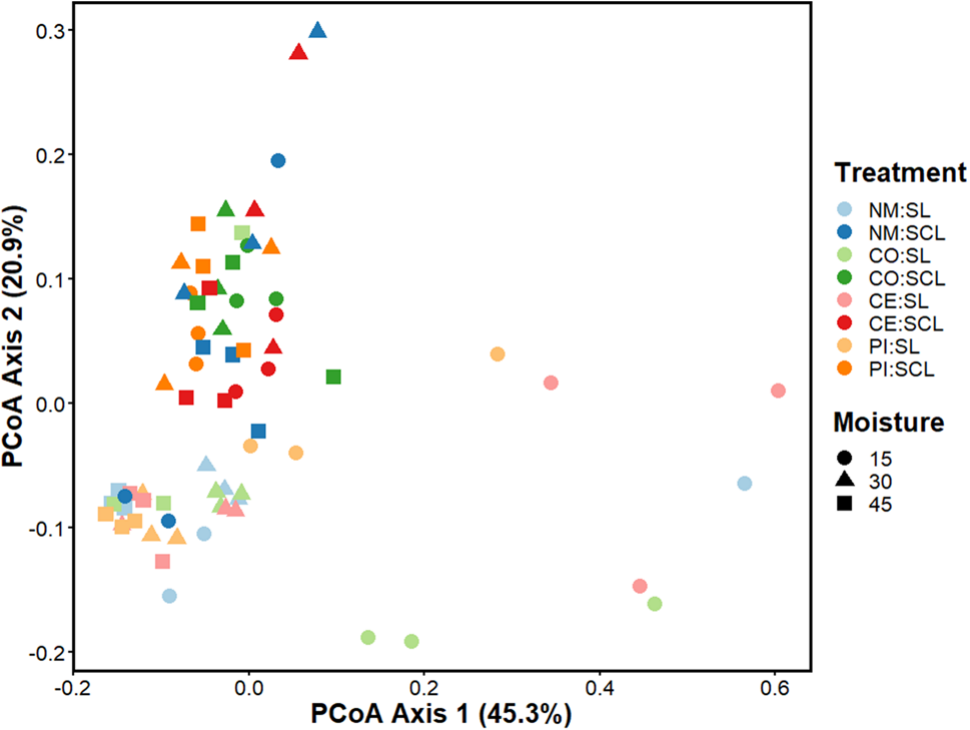
PCoA of ARG abundance profiles after 10-week incubations in a Sandy clay loam (SCL) or sandy loam (SL) soil. Soils were kept at 15, 30, or 45% moisture content, and treatments of no manure (NM), antibiotic-free manure (CO), or manure from cattle administered either cephapirin (CE) or pirlimycin (PI) were added every other week. Shapes represent the moisture content, while colors indicate the treatment and type. Light colors show SCL samples, and darker colors show SL. Significant main effects included moisture (*P* = 0.001) and type (*P* = 0.001), but not manure treatments (*P* = 0.07). Significant interactions included manure treatments * moisture (*P* = 0.03) and moisture * type (*P* = 0.001)

Prior to incubations, many ARGs were already present at detectable levels in the soils and manures used in these experiments (Supplementary Fig. 1). ARG profiles between the two soils had many similarities, but concentrations of several genes such as *vanB*, *qnrB*, *merA*, *copA*, *bla*_*KPC*_, and *aadD* varied by nearly an order of magnitude or more. Among the manure sources, CE was clearly the most different, with elevated concentrations of many ARGs compared to the PI and CO manures, which were much more similar to each other. Following experimental manipulations, multivariate profiles of all ARG abundances varied by soil moisture (*F*_2,71_ = 8.51, *P* = 0.001) and type (*F*_1,71_ = 17.41, *P* = 0.001), but not among manure treatments (F_3,71_ = 1.71, P = 0.07; Fig. 1). The interactions of manure treatments and moisture (*F*_6,71_ = 1.67, *P* = 0.03) and moisture and type (*F*_2,71_ = 9.72, *P* = 0.001) were significant but the interaction of all three main effects was not (*F*_6,71_ = 1.38, *P* = 0.101). Of the main effects and interactions that were significant, the interaction of moisture and type explained the most variation (*R*^2^ = 0.15), followed by type (*R*^2^ = 0.13), moisture (*R*^2^ = 0.13), and the interaction of manure treatments and moisture (*R*^2^ = 0.08).

Post hoc analysis showed that SL and SCL soils had different ARG profiles (*F*_1_ = 9.83, *P* = 0.001). Additionally, the 30 and 45% moisture treatments had different ARG profiles than 15% moisture (*P* < 0.005), but ARG profiles between 30 and 45% moisture were not different (*F*_1_ = 2.34, *P* = 0.060). Within the interactions of moisture and type, all pairwise comparisons were significant (*P* < 0.05), except SCL samples at 15% moisture were not different from SCL samples at 30% moisture (*F*_1_ = 1.89, *P* = 0.089) or 45% (*F*_1_ = 1.30, *P* = 0.228). Pairwise comparisons of the interactions of manure treatments and moisture showed no significant differences once *P* values were adjusted for multiple comparisons.

Changes in specific ARG concentrations can be important regardless of whether the overall profile of ARG concentrations changes, so we also evaluated individual ARGs to provide a more complete picture of changes in the soil. Of the 47 ARGs quantified in this study, 38 varied with moisture content (Supplementary Table 2). Among genes that had significantly different abundances between 15 and 45% moisture, all had higher abundances at 45%. Twenty-seven genes varied with manure treatment (Supplementary Table 3). Generally, genes that varied in abundance with manure treatment tended to have greater abundance in the no manure and pirlimycin treatments compared to the control manure and cephapirin treatments. Thirty-seven genes varied with soil type (Supplementary Table 4); 21 of those had a higher abundance in SL, while 16 had a higher abundance in SCL.

**Fig. 2:**
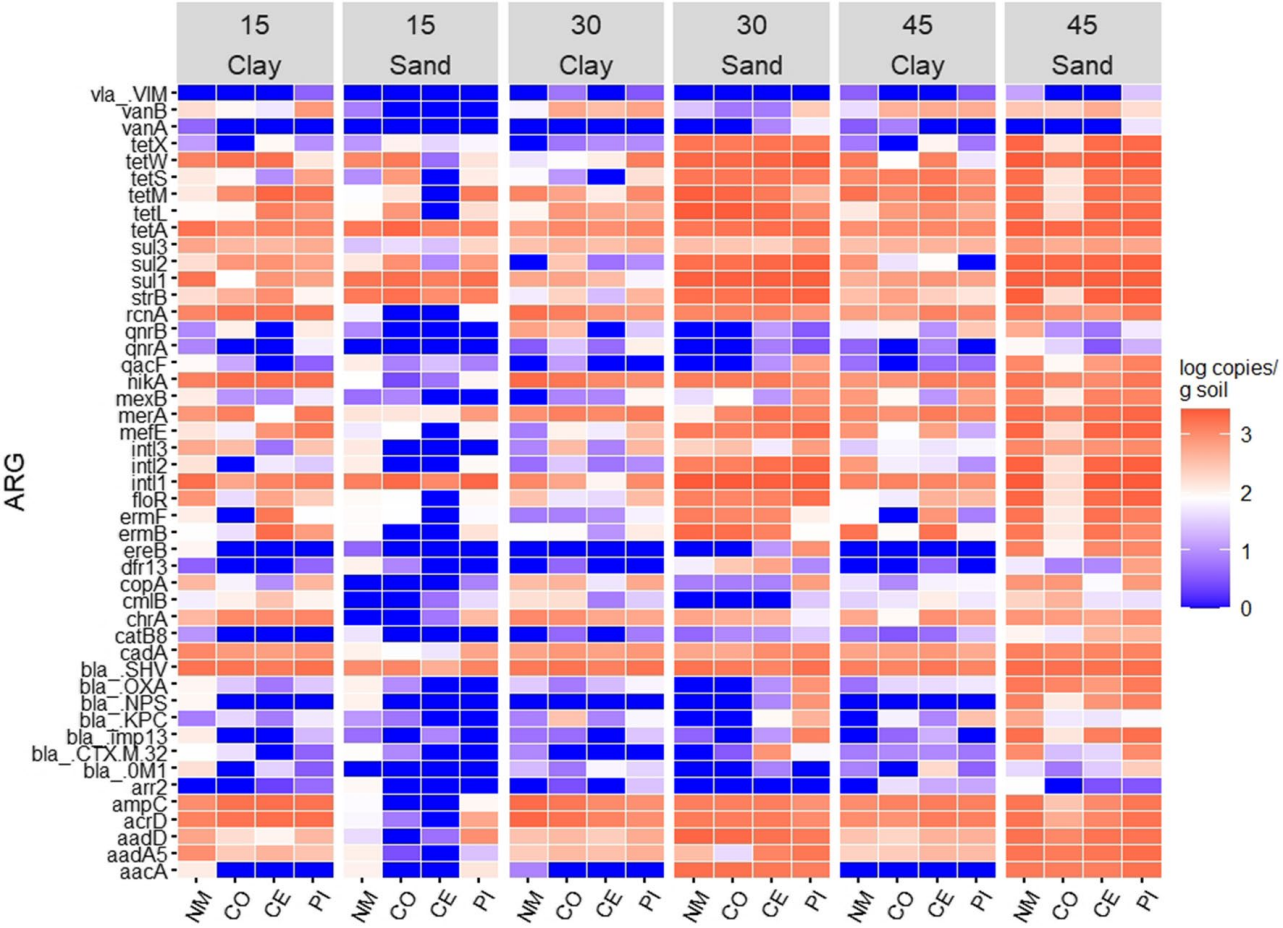
Average abundance (log copies/g soil) of ARGs measured via mfqPCR in soil after 10 weeks in a sandy clay loam (SCL) or sandy loam (SL) soil. Soils were kept at 15, 30, or 45% moisture content; treatments of no manure (NM), antibiotic-free manure (CO), or manure from cattle administered either cephapirin (CE) or pirlimycin (PI) were added every other week

The 10 genes with the highest average abundance across all samples were *bla*_*SHV*_ (3.16 ± 0.15 log copies g^−1^), *tetA* (3.14 ± 0.16 log copies g^−1^), *intI1* (3.10 ± 0.58 log copies g^−1^), *sul1* (2.99 ± 0.60 log copies g^−1^), *merA* (2.93 ± 0.74 log copies g^−1^), *acrD* (2.87 ± 0.83 log copies g^−1^), *nikA* (2.85 ± 0.84 log copies g^−1^), *tetM* (2.83 ± 0.98 log copies g^−1^), *cadA* (2.82 ± 0.51 log copies g^−1^), and *ampC* (2.77 ± 0.96 log copies g^−1^). Eight of the 10 (not *intI1* or *tetA*) most abundant genes varied with moisture (Fig. 2; Supplementary Table 2). Of those that did vary, *bla*_*SHV*_, *acrD, nikA, cadA,* and *ampC* had higher abundances in 30 and 45% moisture than 15% moisture, *su1* and *merA* had higher abundances in 45% moisture than 30 or 15%, and *tetM* was higher at 45% moisture than 15%. All of the most abundant genes except for *tetA* varied with manure treatment (Supplementary Table 3). Four genes (*intI1, acrD, nikA,* and *ampC*) were approximately twice as abundant in no manure and pirlimycin than control manure and cephapirin treatments, while *bla*_*SHV*_ and *tetM* had lower abundances in the cephapirin treatments than all other manure treatments, and *sul1* was more abundant in cephapirin than all other manure treatments. The genes *cadA* and *merA* were most abundant in pirlimycin. All of the 10 most abundant genes except for *merA* varied with type. The genes *bla*_*SHV*_*, acrD, nikA, tetM, cadA,* and *ampC* were more abundant in SCL, while *tetA, intI1,* and *sul1* were more abundant in SL.

### Bacterial communities

**Fig. 3:**
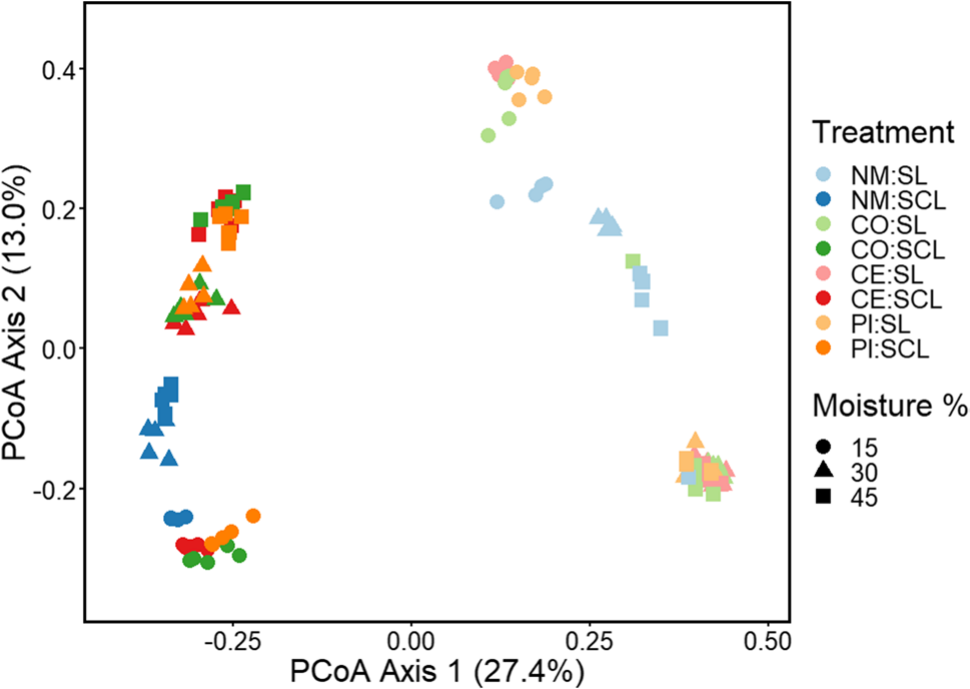
Soil bacterial communities after 10 weeks in a sandy clay loam (SCL) or sandy loam (SL) soil. Soils were kept at 15, 30, or 45% moisture content, and treatments of no manure (NM), antibiotic-free manure (CO), or manure from cattle administered either cephapirin (CE) or pirlimycin (PI) were added every other week. Shapes represent the moisture content; light colors indicate manure treatment in SCL, and dark colors indicate manure treatments in SL. All main effects and interactions were significant (*P* = 0.001)

Changes in soil bacterial community structure were detected as changes in ASVs identified via 16S amplicon sequencing. Bacterial community structure varied across all main effects and interactions (Supplementary Table 5, *P* = 0.001). All post-hoc pairwise comparisons were significantly different (*P* < 0.05), except for the comparison of no manure to control manure in SL at 45% moisture. Given the number of significant pairwise comparisons, it is important to note that not all main effects had an equal impact on bacterial community structure. Soil type explained the most variation (*R*^2^ = 0.25), followed by moisture (*R*^2^ = 0.10), and manure treatment (*R*^2^ = 0.07). Likewise, sample clustering in PCoA was clearly strongest between SCL and SL samples, followed by smaller cluster differences in moisture, and finally manure treatment (Fig. 3). Within each soil type, control treatments with no manure clustered together regardless of moisture content, while the other three treatments clustered by moisture content.

Changes in the bacterial communities were evident at the phylum level (Supplementary Fig. 2), with large changes across treatments in relative abundances of even the most common phyla such as Acidobacteria, Actinobacteria, Bacteroidetes, and Proteobacteria. However, to get a more detailed look at finer resolution at the role of antibiotic exposure, we analyzed changes in relative abundances of genera among the manure treatments (Supplementary Table 6). Indicators for specific treatments included 18 genera for the no manure treatment, 9 genera for the control manure, 4 genera for cephapirin, and 7 genera for pirlimycin. Genera that were indicators for multiple treatments included 3 genera for control manure and cephapirin, 1 genus for cephapirin and pirlimycin, 8 genera for control manure and pirlimycin, and 28 genera for the presence of manure (i.e., control manure, cephapirin, and pirlimycin combined). Of the 18 genera associated with no manure, 3 were from the class Alphaproteobacteria, 3 were from Clostridia, and 3 were from Bacilli. Among the 28 genera associated with manure broadly (control, cephapirin, and pirlimycin), 10 were from Proteobacteria, with 4 in Alphaproteobacteria, 2 in Betaproteobacteria, 2 in Deltaproteobacteria, and 2 in Gammaproteobacteria. Five of the 28 genera were in the class Clostridia, 3 were in Flavobacteria, and 3 were in Saprospirae.

### Respiration

**Fig. 4:**
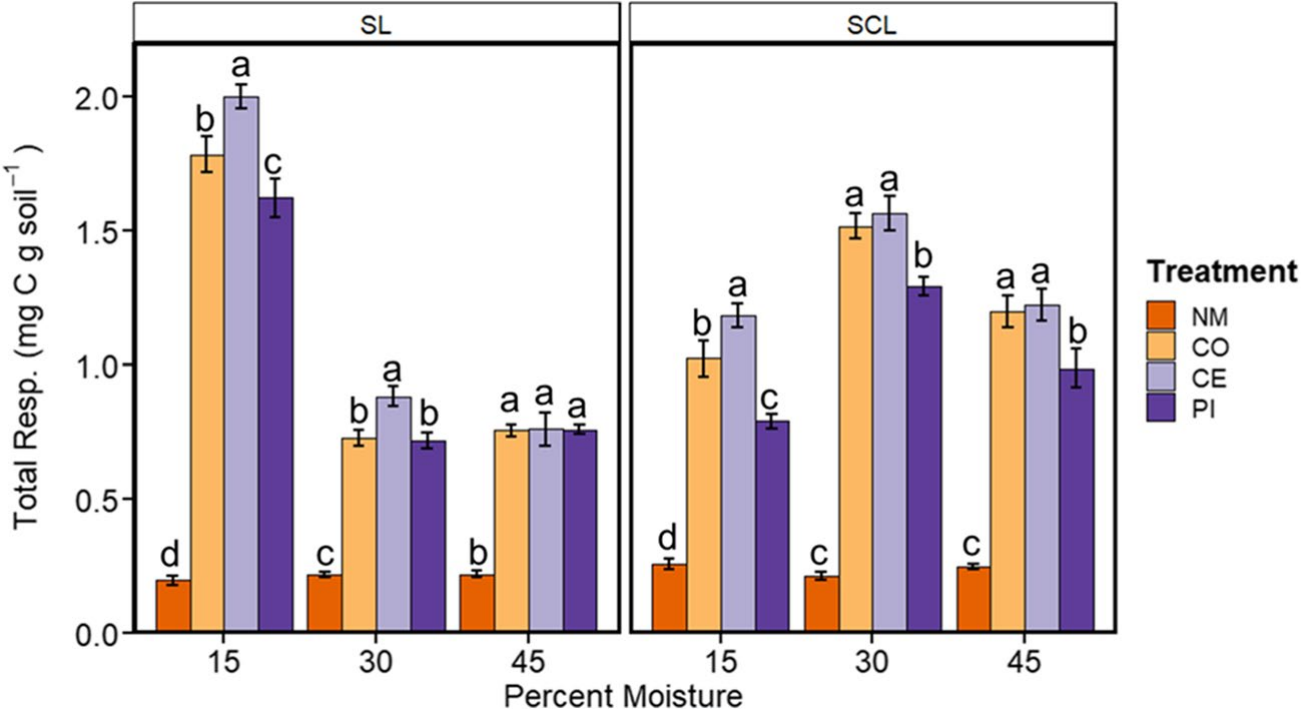
Average ± SE (*n* = 5) cumulative respiration over 10 weeks in a sandy clay loam (SCL) or sandy loam (SL) soil. Soils were kept at 15, 30, or 45% moisture content by mass, and treatments of no manure (NM), antibiotic-free manure (CO), or manure from cattle administered either cephapirin (CE) or pirlimycin (PI) were added every other week. Lowercase letters indicate statistical significance of treatments within moisture and soil type groups

Based on a three-way ANOVA, all main effects and interactions had significant effects on total respiration over the 10-week incubation (Supplementary Table 7, *P* < 0.01). Post hoc analysis showed that soils with manure had, on average, fivefold higher respiration compared to the no manure treatments (Fig. 4). At low moisture content (15%) in both SL and SCL soils, respiration was higher in the cephapirin treatment and lower in the pirlimycin treatment compared to the manure controls (*P* < 0.05). However, those differences disappeared as the moisture content increased. Notably, at 30% moisture in SCL, pirlimycin was 17% lower than the control, while cephapirin was not different from the control. In contrast, in SL at 30%, cephapirin had 20% higher respiration than the control, while pirlimycin was not different. There were no differences in the no manure samples among moisture or type (*P* > 0.05). In SCL samples, manure treatments had the highest respiration at 30% moisture, while the highest respiration in the SL treatments was at 15% moisture. Similar to respiration, all main effects and interactions of three-way ANOVA for active microbial biomass—measured as SIR—were significant at the end of the experiment (Supplementary Table 8, *P* < 0.001; Supplementary Fig. 4). Additionally, in both SIR and respiration, NM tended to be lower while CE tended to have higher SIR than other manure treatments. It is interesting to note patterns of total respiration, and SIR are not similar across treatments. For example, total respiration is highest in the 15% treatment for SL, which is the treatment where SIR is lowest. Similarly, total respiration is highest in the 30% treatment in SCL, where SIR is the lowest.

### Inorganic soil nitrogen pools

**Fig. 5:**
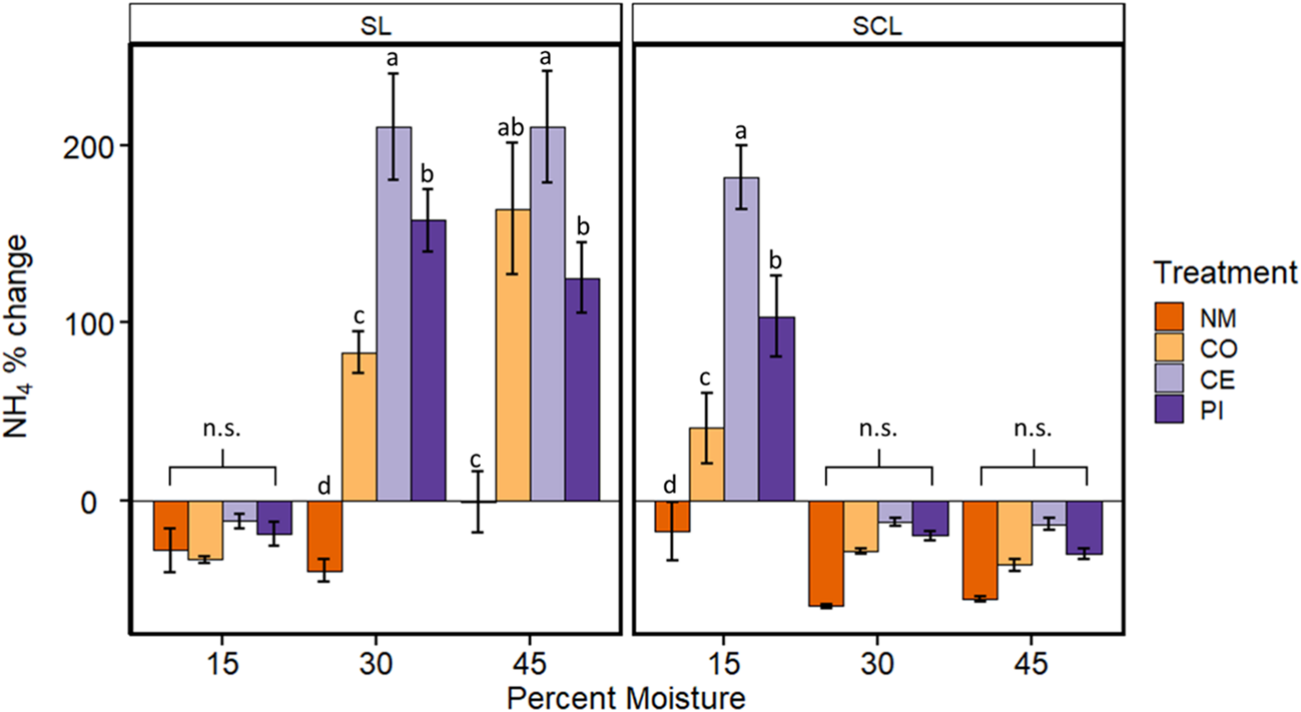
Average ± SE (*n* = 5) percent change in total mass of NH_4_^+^ during experimental incubations of sandy clay loam (SCL) or sandy loam (SL) soils kept at 15, 30, or 45% moisture content (by mass). Treatments of no manure (NM), antibiotic-free manure (CO), or manure from cattle administered either cephapirin (CE) or pirlimycin (PI) were added every other week for 10 weeks. Lowercase letters indicate statistical significance of treatments within moisture and soil type groups. “n.s.” = no significant differences

While there were detectable concentrations of both NO_3_^−^ and NH_4_^+^ in both soils, the amounts in the manure were 1–2 orders of magnitude greater (Supplementary Table 9). Overall, the amount of NO_3_^−^ was similar among the different manures, but the amount of NH_4_^+^ varied greatly, with the greatest amount detected in the CO manure. In the SCL soil, NH_4_^+^ was at 8.05 ± 0.39 mg/kg soil, while SL was at 3.44 ± 0.28 mg/kg soil before any manure additions. Changes in NH_4_^+^ varied based on manure treatment (*F*_*3, 96*_ = 65.44, *P* < 2 × 10^–16^) and type (*F*_*i,96*_ = 93.83, *P* = 7 × 10^–16^), but not moisture (*F*_*2,96*_ = 2.58, P = 0.081). Additionally, all interactions of the 3-way ANOVA were significant (*P* < 0.01). One primary pattern is that the impacts of moisture on NH_4_^+^ change were reversed in the two soil types. For example, in SL, NH4^+^ decreased at 15% moisture with no manure treatment effects and increased at 40 and 45% moisture in treatments with manure added. The reverse was true in SCL soil, with increased NH_4_^+^ and treatment effects at 15% moisture, but none at 30 or 45% moisture (Fig. 5). Across all scenarios, when there were differences in NH_4_^+^ concentrations among treatments, the no manure treatment either decreased or had little change, while the three manure treatments all showed increases. The cephapirin treatment tended to have the largest increase followed by pirlimycin, then the manure control, although in SL at 45% moisture, the control manure was not different from cephapirin or pirlimycin.

**Fig. 6:**
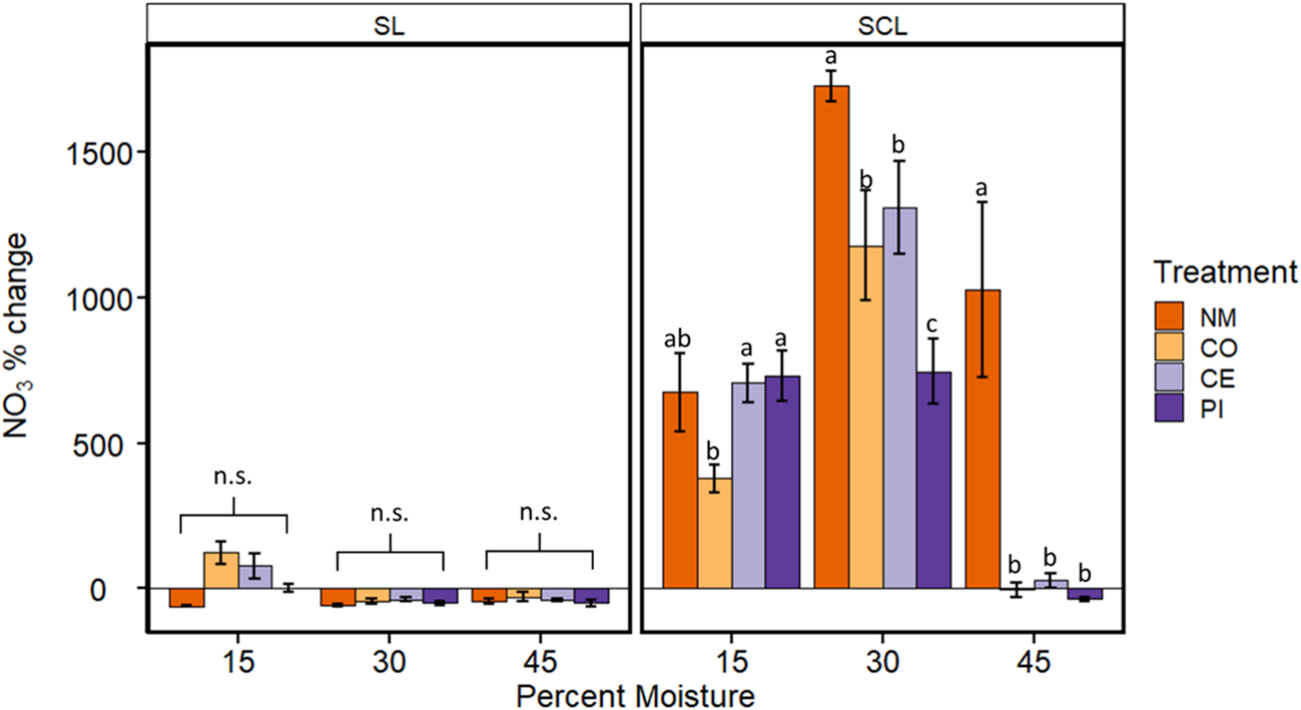
Average ± SE (*n* = 5) percent change in total mass of soil NO_3_^−^ during experimental incubations of sandy clay loam (SCL) or sandy loam (SL) soils kept at 15, 30, or 45% moisture content (by mass). Treatments of no manure (NM), antibiotic-free manure (CO), or manure from cattle administered either cephapirin (CE) or pirlimycin (PI) were added every other week for 10 weeks. Lowercase letters indicate statistical significance of treatments within moisture and soil type groups. “n.s.” = no significant differences

NO_3_^−^ concentrations started at 1.64 ± 0.04 mg/kg soil in SCL and 3.96 ± 0.14 mg/kg soil in SL before any manure additions. Based on the three-way ANOVA, all main effects and interactions were significant (*P* < 0.001), but changes in NO_3_^−^ pools throughout the experiment were quite different between soil types and also from patterns in NH4^+^ described above. In the SL soil, changes in NO_3_^−^ concentration over time were relatively small, and there were no significant differences among treatments at any moisture content (Fig. 6). In the SCL soil, treatments showed large increases from initial concentrations in NO_3_^−^ except at 45% moisture, where the control manure, cephapirin, and pirlimycin treatments showed little to no change from initial concentrations. In contrast to NH_4_^+^, the increase in NO_3_^−^ was highest in the no manure samples, although the no manure treatment was not different compared to any other treatment at 15% moisture.

## Discussion

**Fig. 7:**
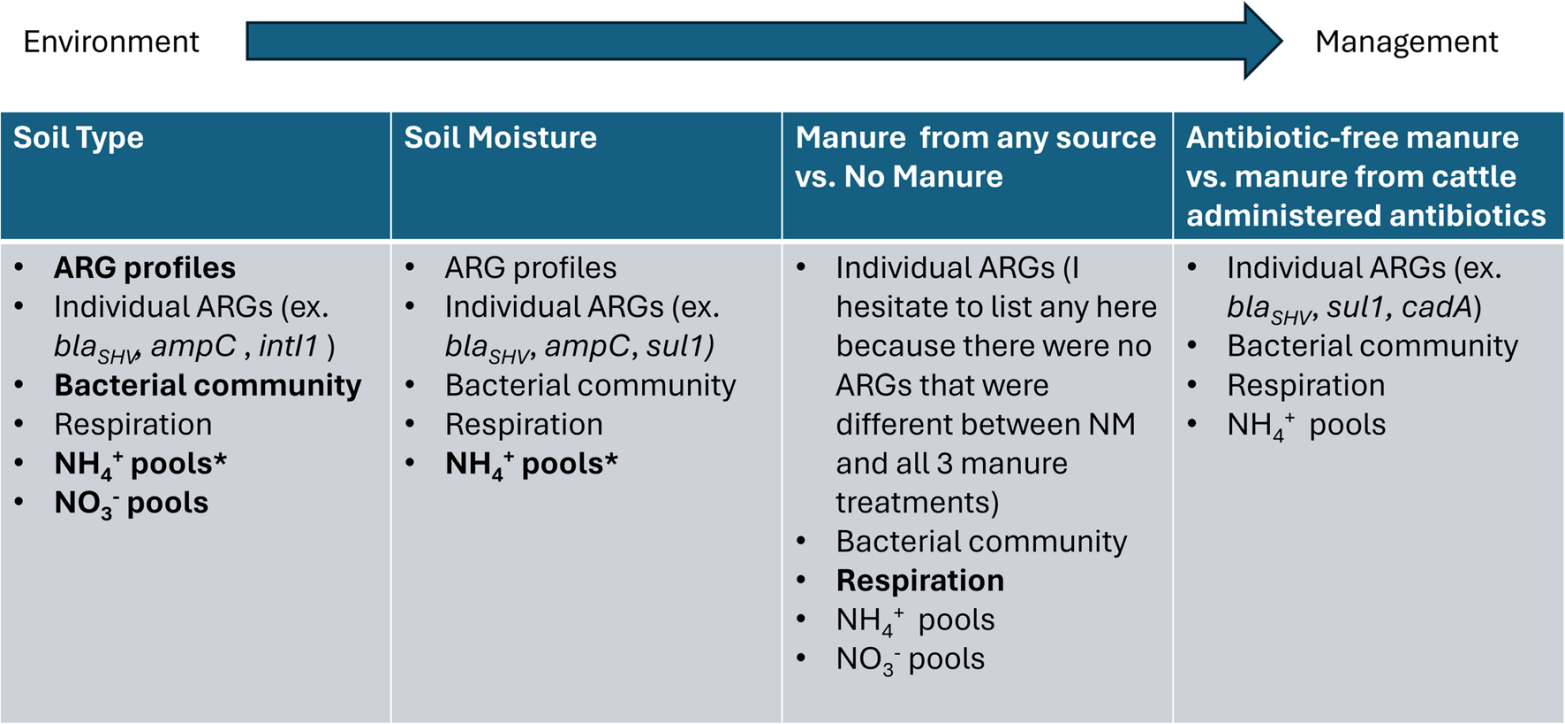
Conceptual diagram of main impacts of soil type, moisture, and additions of manure from cattle administered antibiotics on various aspects of soil microbial communities. Bolded words indicate that the strongest impact was seen under that factor. * indicates the interactive effect of 2 factors had the strongest impact

The purpose of this study was to examine how soil properties, such as moisture and type, influence the response of soil microbial communities to manure from cattle administered antibiotics. Overall, strong effects of soil moisture and type on microbial communities and activity were seen, as expected from previously published work (Dequiedt et al. 2009; Chau et al. 2011; de Vries et al. 2012; Lupatini et al. 2019). A key result of this work, however, was that manure from cattle treated with antibiotics had an impact on microbial community structures, microbial biomass, respiration, and soil N pools that were detectable across a variety of soil moisture and type conditions. Additionally, moisture and soil type showed interactive effects with manure treatments in ARG abundances, highlighting that microbial responses to manure from cattle treated with antibiotics are complex and varied across environmental gradients. Figure 7 summarizes the key findings across the multiple treatments and response variables analyzed in the full experiment.

### Impacts on ARG abundances

The addition of manure alone, regardless of whether cattle had been administered antibiotics, did not impact the overall makeup of ARGs in soil as per multivariate analysis of all individual ARG concentrations. Although multivariate analysis showed an interactive effect of both moisture content and manure treatment on ARG concentrations, post-hoc analysis did not reveal any significant pairwise comparisons. This contrasts with a previous study using a similar approach where we observed higher ARG concentrations in soils with manure compared to no manure controls and no differences between antibiotic-free manure and manure from cattle administered antibiotics (Shawver et al. 2021). Several other studies have shown that manure additions increase ARG concentrations in soil (Fang et al. 2015; Graham et al. 2016; Wepking et al. 2017), and it is unclear why this pattern was not observed here. It is possible that the changes from the manure treatments were too small relative to the effects of soil type and moisture content to detect in this study.

Manure treatments did, however, alter the concentrations of some individual ARGs, including some with relevance to manure and livestock management. Genes that were particularly relevant to this experiment included beta-lactamase genes that convey resistance to cephapirin, macrolide-lincosamide-streptogramin B (MSLb) resistance genes that convey resistance to pirlimycin, and integrase genes, which can indicate potential for horizontal gene transfer. Two beta-lactamase genes (*ampC* and *bla*_*NPS*_) as well as an integrase gene (*intI1*) had higher abundances in pirlimycin and no manure than control manure and cephapirin. Thus, although we expected that manure from cattle treated with cephapirin would lead to an increase of beta-lactamase genes compared to control manure, we observed the opposite. This is despite the fact that the abundances of *intI1* and *ampC* were higher in the manure collected from cattle treated with cephapirin than in the manure collected from cattle treated with pirlimycin (Supplementary Fig. 1. *IntI1* and *ampC* were also present in the original soil before the experiment at concentrations similar to the manure collected from cattle administered cephapirin and no antibiotics, which likely explains why the no manure treatment had high concentrations of those ARGs. *IntI1* is often used as a proxy for antibiotic resistance in soil (Gillings et al. 2015); thus, an increase in *intI1* may indicate an increase in overall antibiotic resistance. Previous work has shown higher abundances of *intI1* in soils with control, pirlimycin, and cephapirin manure compared to no manure (Shawver et al. 2021) and increased *intI1* in clayey soil with manure and tetracycline added (Chessa et al. 2016) Although we chose soils without a known history of manure exposure, these results highlight the importance of considering existing ARG loads when managing agricultural soils.

Beyond manure application, few studies have tested the effects of environmental conditions alone on ARG abundances in soil, and a key finding in this work is that soil moisture and type explained more variation in ARG profiles than manure treatment (e.g., Fig. 1). Five beta-lactamase genes (*ampC, bla*_*NPS,*_* bla*_*OXA*_*, bla*_*SHV*_, and *bla*_*VIM*_), 1 MSLb gene (*ermB*), and 2 integrase genes (*intl2* and *intl3*) had higher abundances at 45% moisture compared to 15% moisture, indicating that higher moisture contents may promote increased AMR in soils. Furthermore, 6 beta-lactamase genes (*ampC, bla*_*CTX-M-32*_*, bla*_*imp13*_*, bla*_*NPS*_*, bla*_*SHV*_, and *bla*_*VIM*_), 1 MSLb gene (*ermF*), and 2 integrase genes (*intI1* and *intl2*) varied with soil type. Among the genes that varied with soil type, three (*ampC, bla*_*SHV*_*,* and *bla*_*VIM*_) had higher abundances in SCL, while the rest had higher abundances in SL. Of the ARGs that were different among soil type, *bla*_*VIM*_, was slightly higher in the SCL soil prior to the start of the experiment, while *ermF* was present in SL but not SCL (Supplementary Fig. 1). The remaining ARGs that had different ARG concentrations had similar background concentrations in the two soils, indicating that there was a change in ARG abundances over the course of the experiment. Previous work has shown soil texture can influence abundances of individual ARGs after exposure to antibiotics, but the results have been equivocal, with some reports of larger impacts of ARGs in soils with finer textures (Chander et al. 2005) and others finding larger impacts from coarser textures (Pankow 2017; Blau et al. 2018; Chen et al. 2018). The findings from this study may explain the discrepancies from previous works, as we found that soil type impacts numerous ARG abundances in response to antibiotic exposure; however, the direction of impact varies with individual genes.

The mechanisms of how soil interacts with AMR within the soil microbiome remain difficult to disentangle, however, and will require considerable future research. As noted in the methods, the soils used in this study varied in many properties, not just texture. Thus, while we were able to document the importance of soil type in mediating responses to manure treatments, it is difficult to pinpoint whether differences in texture or other properties are the main cause. For example, it is possible that pH may be at least partially responsible for differences, as previous work has shown that pH can impact ARG abundances in manured soils (Xiao et al. 2016). Regardless, it is further evidence that environmental conditions, as well as the interactions of those conditions with antibiotic and manure management, clearly play important roles in mitigating environmental persistence and transmission of ARGs.

### Impacts on bacterial community structure

Bacterial community structure varied with soil moisture, type, and manure treatments, as well as their interactions. Soil type explained the most variation in bacterial communities, and moisture had the next largest impact. However, these results were unsurprising as soil microbial community structure is well known to respond to both texture (Dequiedt et al. 2009; Roberts et al. 2011; Chau et al. 2011; de Vries et al. 2012) and moisture (de Vries et al. 2012; Lupatini et al. 2019). Furthermore, the two soils clearly contained different microbial communities at the beginning of the experiment (Supplementary Figs. 3 and 5). The important result of this work was that manure treatment had detectable effects across these ranges of soil conditions. While manure treatment explained less variability than soil type and moisture, both the application of manure and whether manure was from cattle given antibiotics resulted in measurable changes in microbial community structure. The biggest changes resulted from manure addition (i.e., between the microcosms with no manure and all 3 of the microcosms with manure additions). This is likely due to the influx of organic carbon and other nutrients present in manure, and many studies have shown that nutrients influence microbial community structure (Lauber et al. 2008; de Vries et al. 2012; Chávez-Romero et al. 2016). Of the 18 bacterial genera associated with no manure, some are common soil bacteria, including *Variovorax* and *Paenibacillus* (Delgado-Baquerizo et al. 2018), while others have known important ecological functions, such as the N-fixers *Phormidium* (Berrendero et al. 2016), *Nostoc* (Lindberg et al. 2004), *Leptolyngbya* (Tsujimoto et al. 2016), *Brevibacillus* (Nehra et al. 2016), and *Desulfosporosinus*, which can also reduce sulfur (Thajudeen et al. 2017). These associated genera were negatively impacted by additions of manure and manure with antibiotics.

Among the genera with increased relative abundances in soils amended with manure, several are common gut bacteria, including *Ruminococcus* (cephapirin and control manure treatments), *Turicibacter* (pirlimycin and control manure treatments), and *Coprococcus* (cephapirin, pirlimycin, and control manure treatments). There were also several pathogen-containing genera associated with soils amended with manure, including *Corynebacterium* (control manure treatments), *Clostridium* and *Treponema* (pirlimycin manure treatments), *Cellulosimicrobium* (cephapirin and control manure treatments), *Dietzia* and *Facklamia,* (pirlimycin and control manure treatments), and *Acinetobacter* (control, cephapirin, and pirlimycin manure treatments). High abundances of *Acinetobacter* have been found previously in soils with a long history of manure applications (Wepking et al. 2017), and the genus is of particular importance as containing human pathogens that are increasingly antibiotic resistant (Manchanda et al. 2010). Furthermore, several genera that were more abundant in soils amended with manure from cattle treated with antibiotics have been found previously to be carriers for ARGs, including *Formivibrio* (Jiang et al. 2021) in cephapirin manure treatments, *Stenotrophomonas* (Ryan et al. 2009) in pirlimycin manure treatments, *Facklamia* (Rahmati et al. 2017) in control and pirlimycin, and *Pedobacter* (Bjerketorp et al. 2021) in control, cephapirin, and pirlimycin manure treatments. Although we did not find an increase in individual ARG concentrations in all manure treatments, associations of taxa potentially containing pathogens and ARG carriers represent other possible risks to human health that should be considered across a range of soil conditions.

### Impacts on microbial function

While human and animal health risks are understandably a major area of focus in managing agricultural antibiotic usage, recent evidence suggests that changes to soil microbial communities following exposure to manure containing antibiotics can also impact biogeochemistry and ecosystem fluxes (Wepking et al. 2019). While we saw increased respiration in soils with manure added, this was, again, not surprising since organic carbon additions are known to increase heterotrophic activity (Šantrůčková et al. 2003). However, other results from this study further document an effect of manure from cattle administered antibiotics specifically, with cephapirin manure treatments having higher respiration. As previously stated, concentrations of antibiotic compounds in soils were not quantified in this study, and so the actual mechanisms of the effects are not known in this study. However, some results of changes in the soil biogeochemistry are consistent with known modes of action of different antibiotics. For example, increased fungal growth was visually obvious in cephapirin-amended microcosms, which agrees with the bactericidal effect of cephapirin and could affect respiration rates. Higher metabolic cost for maintenance of AMR has also been raised as a possibility (Wepking et al. 2017), although we did not see increased respiration rates in the pirlimycin manure treatment. It is possible that pirlimycin, as a bacteriostatic antibiotic, was simply slowing growth and inhibiting overall respiration. However, this is the opposite of what was previously observed by Wepking et al. (2019), highlighting the need for additional controlled work investigating antibiotic effects on soil respiration.

Beyond carbon cycling, we also quantified dissolved inorganic soil N pools across microcosms. NH_4_^+^ concentrations generally increased in manure treatments where respiration was lower (SCL at 15%, SL at 30 and 45%) and decreased where respiration was higher. Nitrate concentrations were also strongly affected by soil type and moisture, and previous studies have demonstrated N cycling processes responding to soil texture (Šantrůčková et al. 2003; Enwall et al. 2010; Morales et al. 2015), moisture content (Morugán-Coronado et al. 2019), and available nutrients such as carbon (Šantrůčková et al. 2003; Deslippe et al. 2014). Among manure treatments, the largest NH_4_^+^ increases were in cephapirin manure treatments, followed by pirlimycin. This would suggest potentially decreased nitrification rates, and antibiotics are known to inhibit nitrification (Tomlinson et al. 1966; Klaver and Matthews 1994; Kotzerke et al. 2008; Toth et al. 2011). For NO_3_^−^, the effects of manure treatment within the SCL soils were mixed. However, the smaller increase in NO_3_^−^ in cephapirin and pirlimycin manure treatments compared to no manure at higher moisture contents suggests that denitrification was stimulated by antibiotics, which agrees with previous studies (Ahmad et al. 2014; D’Alessio et al. 2019). It is important to note this experiment was not designed to parse the many possible biogeochemical fluxes that impact N pools in soil ecosystems. However, the overall treatment differences observed here indicate that antibiotic usage in cattle may alter how manure impacts soil biogeochemistry and the availability of important plant nutrients, and those effects are mediated by the soil environment.

### Conclusions

Overall, soil conditions played an important role in the impact of manure from cattle administered antibiotics on soil microbial communities. Specifically, type, followed by moisture, had the greatest impact on bacterial community structure, while the addition of manure had a smaller but still significant impact across a range of soil conditions. Additionally, moisture had a strong impact on overall ARG abundances, even in the absence of manure from antibiotic-treated cattle. While an important result of this work is demonstrating the effects of manure from cattle administered antibiotics across a variety of soil conditions, there are two other key findings. Firstly, different antibiotics can influence the impact of antibiotic exposure. With manure from cattle treated with cephapirin, we generally observed higher respiration, greater accumulation of NH_4_^+^, and lower ARG abundances than in soil treated with manure from cattle administered pirlimycin. And secondly, soil conditions can interact with manure exposure and alter the responses of soil communities, making it clear that the soil environment is important to consider in managing soil exposure to manure and antibiotics. Notably, higher moisture content corresponds with higher ARG abundances, suggesting management strategies should potentially avoid manure applications in wetter conditions when possible. Further work is clearly needed to fully understand the interactions among the soil environment, manure and antibiotic management, and antimicrobial resistance in soil.

### Supplementary Information

Below is the link to the electronic supplementary material.

Supplementary file1 (DOCX 667 KB)
