# Supplementary material for: Soil type and moisture content alter soil microbial responses to manure from cattle administered antibiotics
Source: Environ Sci Pollut Res Int. 2024 Mar 20;31(18):27259–72. doi: 10.1007/s11356-024-32903-z (PMC11052774; doi:10.1007/s11356-024-32903-z)
Supplement: Supplementary file 1 — Supplementary file1 (DOCX 667 KB) [file 11356_2024_32903_MOESM1_ESM.docx]

# Soil type and moisture content alter soil microbial responses to antibiotic and manure exposure

Environmental Science and Pollution Research

Sarah Shawver^a*^, Satoshi Ishii, Michael S. Strickland, Brian Badgley

^a^School of Plant and Environmental Sciences, Virginia Tech, Blacksburg, VA 24061, USA

*Corresponding author. Email: [sarahes@vt.edu](mailto:sarahes@vt.edu)

# Supplementary Methods

## SIR

A 2 g aliquot of soil from each mesocosm was incubated in a 50 ml conical centrifuge tube at 20ºC overnight. Four ml of autolyzed yeast (99 g/ml) was then added to each tube. Tubes were then shaken for 1 hour, tightly capped, flushed with CO_2_-free air and incubated for 5 hours. After 5 hours, CO_2_ concentrations were then measured using an IRGA as described for respiration.

# Supplementary Results

**Supplementary Table 1.** Soil testing data from the two soil types.

| Soil | pH | BpH | P ppm | K ppm | Ca ppm | Mg ppm | Zn ppm | Mn ppm | Cu ppm | Fe ppm | B ppm | % OM | CEC meq/100g | % Base Sat | % Ca Sat | % Mg Sat | % K Sat |
| --- | --- | --- | --- | --- | --- | --- | --- | --- | --- | --- | --- | --- | --- | --- | --- | --- | --- |
| SCL | 6.26 | 6.23 | 3 | 57 | 497 | 184 | 2.1 | 13.1 | 0.8 | 15.5 | 0.3 | 3.8 | 5.2 | 80.4 | 48.2 | 29.4 | 2.8 |
| SL | 5.8 | 6.25 | 42 | 94 | 319 | 48 | 1.1 | 15.3 | 0.9 | 13.1 | 0.1 | 0.8 | 3.1 | 71.4 | 51.1 | 12.6 | 7.7 |

**Supplementary Table 2.** Aligned rank ANOVA with FDR-adjusted p-values and average gene abundances for each moisture content. Lowercase letters indicate significant differences for post-hoc analyses.

|  | **Aligned rank ANOVA** | | **Average ± se log copies/g soil** | | | | | |
| --- | --- | --- | --- | --- | --- | --- | --- | --- |
| **Gene** | **F-value** | **Adj. P-value** | **15%** | | **30%** | | **45%** | |
| *aacA* | 23.70 | **4.66E-07** | 0.82 ± 0.81 | c | 1.72 ± 0.93 | a | 1.59 ± 0.94 | b |
| *aadA5* | 39.63 | **3.23E-09** | 1.89 ± 0.7 | c | 2.58 ± 0.37 | b | 2.85 ± 0.23 | a |
| *aadD* | 27.57 | **8.38E-08** | 1.95 ± 0.65 | b | 2.9 ± 0.23 | a | 2.86 ± 0.23 | a |
| *acrD* | 23.08 | **5.54E-07** | 2.38 ± 0.76 | b | 3.14 ± 0.08 | a | 3.06 ± 0.16 | a |
| *ampC* | 27.98 | **8.29E-08** | 2.16 ± 0.85 | b | 3.11 ± 0.09 | a | 3.03 ± 0.2 | a |
| *arr2* | 10.34 | **5.74E-04** | 0.39 ± 0.54 | b | 0.23 ± 0.37 | c | 0.86 ± 0.57 | a |
| *bla_NDM1_* | 3.47 | 9.60E-02 | 0.56 ± 0.57 |  | 0.79 ± 0.67 |  | 1.25 ± 0.69 |  |
| *bla_CTX-M32_* | 1.63 | **4.82E-02** | 0.91 ± 0.75 | a | 0.78 ± 0.73 | a | 1.55 ± 0.82 | a |
| *bla_imp13_* | 9.07 | 2.26E-01 | 0.66 ± 0.67 |  | 0.96 ± 0.75 |  | 1.71 ± 0.89 |  |
| *bla_KPC_* | 2.61 | **1.02E-03** | 0.88 ± 0.72 | a | 1.34 ± 0.8 | a | 1.65 ± 0.76 | a |
| *bla_NPS_* | 33.40 | **1.92E-08** | 0.52 ± 0.67 | b | 0.48 ± 0.64 | c | 1.4 ± 0.9 | a |
| *bla_OXA_* | 9.45 | **8.59E-04** | 1.13 ± 0.78 | b | 1.17 ± 0.75 | b | 2.24 ± 0.71 | a |
| *bla_SHV_* | 10.06 | **6.20E-04** | 3.09 ± 0.12 | b | 3.18 ± 0.05 | a | 3.19 ± 0.07 | a |
| *bla_VIM_* | 9.17 | **9.93E-04** | 0.08 ± 0.22 | b | 0.16 ± 0.32 | a | 0.47 ± 0.48 | a |
| *cadA* | 8.61 | **1.36E-03** | 2.62 ± 0.49 | b | 2.87 ± 0.09 | a | 2.95 ± 0.09 | a |
| *catB8* | 8.18 | **1.79E-03** | 0.35 ± 0.54 | b | 0.68 ± 0.64 | b | 1.55 ± 0.73 | a |
| *chrA* | 22.86 | **5.54E-07** | 1.97 ± 0.79 | b | 2.67 ± 0.35 | a | 2.76 ± 0.35 | a |
| *cmlB* | 6.58 | **5.12E-03** | 1.39 ± 0.67 | ab | 1.03 ± 0.67 | b | 1.91 ± 0.6 | a |
| *copA* | 6.00 | **7.63E-03** | 1.16 ± 0.78 | b | 1.88 ± 0.71 | ab | 2.1 ± 0.72 | a |
| *dfr13* | 3.78 | **3.89E-02** | 0.55 ± 0.63 | b | 1.07 ± 0.76 | a | 0.87 ± 0.73 | ab |
| *ereB* | 29.08 | **6.26E-08** | 0.34 ± 0.54 | c | 0.5 ± 0.66 | b | 1.39 ± 0.9 | a |
| *ermB* | 5.42 | **1.10E-02** | 1.8 ± 0.88 | b | 2.32 ± 0.8 | ab | 2.74 ± 0.62 | a |
| *ermF* | 0.95 | 3.95E-01 | 1.67 ± 0.87 |  | 1.97 ± 0.83 |  | 2.17 ± 0.83 |  |
| *floR* | 5.39 | **1.10E-02** | 2.03 ± 0.69 | b | 2.61 ± 0.5 | a | 2.63 ± 0.62 | a |
| *intI1* | 1.23 | 3.13E-01 | 3.15 ± 0.14 |  | 3.06 ± 0.4 |  | 3.1 ± 0.4 |  |
| *IntI2* | 10.26 | **5.74E-04** | 1.22 ± 0.84 | c | 2.09 ± 0.82 | b | 2.45 ± 0.76 | a |
| *IntI3* | 7.27 | **3.29E-03** | 1.39 ± 0.8 | b | 2.05 ± 0.64 | ab | 2.33 ± 0.63 | a |
| *mefE* | 3.31 | 5.43E-02 | 2.04 ± 0.81 |  | 2.49 ± 0.61 |  | 2.63 ± 0.64 |  |
| *merA* | 5.85 | **8.35E-03** | 2.66 ± 0.62 | b | 2.96 ± 0.37 | b | 3.15 ± 0.11 | a |
| *mexB* | 9.84 | **6.84E-04** | 0.96 ± 0.79 | b | 1.41 ± 0.84 | b | 2.51 ± 0.67 | a |
| *nikA* | 10.44 | **5.74E-04** | 2.34 ± 0.77 | b | 3.12 ± 0.09 | a | 3.07 ± 0.11 | a |
| *qacF* | 7.63 | **2.59E-03** | 1.17 ± 0.77 | b | 0.62 ± 0.65 | b | 1.63 ± 0.83 | a |
| *qnrA* | 1.57 | 2.33E-01 | 0.35 ± 0.46 |  | 0.76 ± 0.59 |  | 0.85 ± 0.68 |  |
| *qnrB* | 3.75 | **3.89E-02** | 0.79 ± 0.73 | a | 1.05 ± 0.71 | ab | 1.68 ± 0.78 | b |
| *rcnA* | 30.17 | **5.12E-08** | 2.14 ± 0.84 | b | 3.05 ± 0.12 | a | 3 ± 0.14 | a |
| *strB* | 2.12 | 1.47E-01 | 2.8 ± 0.53 |  | 2.65 ± 0.55 |  | 2.79 ± 0.49 |  |
| *sul1* | 6.55 | **5.12E-03** | 2.96 ± 0.39 | b | 2.93 ± 0.42 | b | 3.09 ± 0.18 | a |
| *sul2* | 5.73 | **8.92E-03** | 2.58 ± 0.61 | a | 2.17 ± 0.84 | b | 2.49 ± 0.77 | a |
| *sul3* | 12.59 | **1.45E-04** | 2.27 ± 0.44 | c | 2.57 ± 0.11 | b | 2.71 ± 0.1 | a |
| *tetA* | 2.83 | 8.09E-02 | 3.14 ± 0.08 |  | 3.11 ± 0.1 |  | 3.17 ± 0.1 |  |
| *tetL* | 4.88 | **1.58E-02** | 2.09 ± 0.83 | b | 2.94 ± 0.41 | a | 2.87 ± 0.53 | a |
| *tetM* | 7.14 | **3.48E-03** | 2.46 ± 0.78 | b | 2.94 ± 0.42 | ab | 3.07 ± 0.39 | a |
| *tetS* | 18.09 | **6.58E-06** | 1.8 ± 0.86 | b | 2.23 ± 0.78 | b | 3.03 ± 0.38 | a |
| *tetW* | 5.00 | **1.47E-02** | 2.59 ± 0.72 | b | 2.77 ± 0.64 | ab | 2.92 ± 0.54 | a |
| *tetX* | 0.95 | 3.95E-01 | 1.37 ± 0.86 |  | 1.93 ± 0.89 |  | 1.96 ± 0.91 |  |
| *vanA* | 13.03 | **1.18E-04** | 0.08 ± 0.23 | b | 0.33 ± 0.52 | a | 0.39 ± 0.52 | a |
| *vanB* | 15.36 | **2.97E-05** | 1.26 ± 0.76 | c | 1.92 ± 0.67 | b | 2.44 ± 0.34 | a |

**Supplementary Table 3.** Aligned rank ANOVA with FDR-adjusted p-values and average gene abundances for each treatment. Lowercase letters indicate significant differences for post-hoc analyses.

| **Aligned rank ANOVA** | | | **Average ± se log copies/g soil** | | | | | | | |
| --- | --- | --- | --- | --- | --- | --- | --- | --- | --- | --- |
| **Gene** | **F.value** | **Adjusted P-value** | **NM** | | **CO** | | **CE** | | **PI** | |
| *aacA* | 14.58 | **5E-06** | 2.02 ± 0.89 | a | 1.06 ± 0.89 | b | 1.05 ± 0.88 | b | 1.42 ± 0.94 | a |
| *aadA5* | 11.40 | **5E-05** | 2.75 ± 0.24 | a | 2.11 ± 0.61 | b | 2.34 ± 0.64 | b | 2.6 ± 0.44 | a |
| *aadD* | 15.42 | **5E-06** | 2.82 ± 0.28 | a | 2.25 ± 0.65 | b | 2.38 ± 0.57 | b | 2.89 ± 0.18 | a |
| *acrD* | 17.47 | **2E-06** | 3.11 ± 0.12 | a | 2.68 ± 0.6 | b | 2.62 ± 0.7 | b | 3.06 ± 0.13 | a |
| *ampC* | 14.97 | **5E-06** | 3.09 ± 0.12 | a | 2.52 ± 0.71 | b | 2.6 ± 0.69 | b | 2.91 ± 0.43 | a |
| *arr2* | 3.15 | 0.0557 | 0.68 ± 0.66 |  | 0.35 ± 0.48 |  | 0.34 ± 0.39 |  | 0.63 ± 0.54 |  |
| *bla_NDM1_* | 1.12 | **0.0203** | 1.06 ± 0.69 | a | 0.26 ± 0.44 | a | 1.32 ± 0.71 | a | 0.85 ± 0.65 | a |
| *bla_CTX-M-32_* | 3.61 | 0.3641 | 1.52 ± 0.86 |  | 0.9 ± 0.72 |  | 0.9 ± 0.77 |  | 1.04 ± 0.79 |  |
| *bla_imp13_* | 1.70 | **0.0345** | 1.31 ± 0.86 | a | 0.59 ± 0.67 | a | 1.06 ± 0.82 | a | 1.52 ± 0.85 | a |
| *bla_KPC_* | 4.27 | 0.2337 | 0.97 ± 0.79 |  | 1.39 ± 0.75 |  | 1.04 ± 0.78 |  | 1.76 ± 0.75 |  |
| *bla_NPS_* | 19.38 | **1E-06** | 1.25 ± 0.89 | a | 0.35 ± 0.59 | b | 0.64 ± 0.71 | b | 1.01 ± 0.85 | a |
| *bla_OXA_* | 1.46 | 0.2923 | 1.66 ± 0.85 |  | 1.31 ± 0.8 |  | 1.27 ± 0.78 |  | 1.85 ± 0.77 |  |
| *bla_SHV_* | 5.70 | **0.0053** | 3.19 ± 0.05 | a | 3.17 ± 0.07 | a | 3.08 ± 0.12 | b | 3.19 ± 0.08 | a |
| *bla_VIM_* | 5.46 | **0.0064** | 0.31 ± 0.4 | a | 0.13 ± 0.32 | ab | 0 ± 0 | b | 0.52 ± 0.5 | a |
| *cadA* | 6.66 | **0.0027** | 2.92 ± 0.11 | b | 2.71 ± 0.4 | b | 2.72 ± 0.4 | b | 2.91 ± 0.09 | a |
| *catB8* | 1.23 | 0.3312 | 1.09 ± 0.79 |  | 0.64 ± 0.63 |  | 0.72 ± 0.69 |  | 1.05 ± 0.71 |  |
| *chrA* | 7.42 | **0.0014** | 2.49 ± 0.58 | bc | 2.25 ± 0.72 | c | 2.49 ± 0.54 | b | 2.67 ± 0.41 | a |
| *cmlB* | 1.26 | 0.328 | 1.38 ± 0.7 |  | 1.44 ± 0.69 |  | 1.3 ± 0.71 |  | 1.65 ± 0.63 |  |
| *copA* | 3.85 | **0.0309** | 1.87 ± 0.72 | ab | 1.52 ± 0.81 | b | 1.19 ± 0.8 | b | 2.3 ± 0.62 | a |
| *dfr13* | 1.65 | 0.2421 | 1.08 ± 0.79 |  | 0.81 ± 0.69 |  | 0.73 ± 0.7 |  | 0.73 ± 0.71 |  |
| *ereB* | 14.79 | **5E-06** | 1.01 ± 0.83 | a | 0.33 ± 0.56 | b | 0.68 ± 0.76 | b | 1 ± 0.84 | a |
| *ermB* | 2.31 | 0.1261 | 2.72 ± 0.61 |  | 1.82 ± 0.89 |  | 2.31 ± 0.85 |  | 2.35 ± 0.76 |  |
| *ermF* | 1.99 | 0.1772 | 2.31 ± 0.78 |  | 1.32 ± 0.88 |  | 2.23 ± 0.83 |  | 1.92 ± 0.82 |  |
| *floR* | 3.64 | **0.0345** | 2.78 ± 0.45 | a | 2.03 ± 0.73 | ab | 2.23 ± 0.73 | b | 2.69 ± 0.46 | a |
| *intI1* | 9.14 | **0.0003** | 3.26 ± 0.1 | a | 2.94 ± 0.46 | b | 3 ± 0.45 | b | 3.22 ± 0.11 | a |
| *IntI2* | 2.80 | 0.0781 | 2.53 ± 0.72 |  | 1.42 ± 0.87 |  | 1.79 ± 0.87 |  | 2.01 ± 0.87 |  |
| *IntI3* | 2.57 | 0.096 | 2.23 ± 0.66 |  | 2.05 ± 0.66 |  | 1.33 ± 0.8 |  | 2.13 ± 0.69 |  |
| *mefE* | 1.31 | 0.3236 | 2.49 ± 0.71 |  | 2.15 ± 0.73 |  | 2.32 ± 0.75 |  | 2.6 ± 0.62 |  |
| *merA* | 3.64 | **0.0345** | 2.9 ± 0.45 | b | 2.92 ± 0.43 | ab | 2.78 ± 0.59 | b | 3.12 ± 0.11 | a |
| *mexB* | 1.38 | 0.3126 | 1.83 ± 0.83 |  | 1.46 ± 0.87 |  | 1.17 ± 0.87 |  | 2.09 ± 0.78 |  |
| *nikA* | 7.69 | **0.0012** | 3.1 ± 0.11 | a | 2.67 ± 0.63 | b | 2.73 ± 0.59 | b | 2.92 ± 0.43 | a |
| *qacF* | 1.28 | 0.3276 | 1.38 ± 0.88 |  | 0.85 ± 0.7 |  | 0.99 ± 0.75 |  | 1.35 ± 0.82 |  |
| *qnrA* | 2.81 | 0.0781 | 0.72 ± 0.68 |  | 0.5 ± 0.57 |  | 0.49 ± 0.55 |  | 0.93 ± 0.58 |  |
| *qnrB* | 4.71 | **0.0131** | 1.62 ± 0.81 | a | 1.27 ± 0.78 | a | 0.48 ± 0.56 | b | 1.38 ± 0.76 | a |
| *rcnA* | 12.90 | **2E-05** | 3.03 ± 0.13 | a | 2.54 ± 0.7 | b | 2.54 ± 0.68 | b | 2.85 ± 0.42 | a |
| *strB* | 0.02 | 0.9957 | 2.71 ± 0.63 |  | 2.76 ± 0.46 |  | 2.74 ± 0.52 |  | 2.78 ± 0.5 |  |
| *sul1* | 6.03 | **0.0045** | 3.11 ± 0.19 | a | 2.92 ± 0.45 | b | 3.05 ± 0.18 | a | 2.91 ± 0.45 | b |
| *sul2* | 5.07 | **0.0093** | 2.46 ± 0.82 | ab | 2.76 ± 0.45 | a | 2.2 ± 0.83 | b | 2.22 ± 0.84 | b |
| *sul3* | 5.73 | **0.0053** | 2.59 ± 0.16 | b | 2.44 ± 0.36 | b | 2.4 ± 0.38 | b | 2.66 ± 0.11 | a |
| *tetA* | 0.34 | 0.8116 | 3.14 ± 0.12 |  | 3.16 ± 0.08 |  | 3.13 ± 0.09 |  | 3.14 ± 0.07 |  |
| *tetL* | 1.81 | 0.2117 | 2.42 ± 0.81 |  | 2.72 ± 0.59 |  | 2.59 ± 0.7 |  | 2.83 ± 0.45 |  |
| *tetM* | 6.20 | **0.004** | 3 ± 0.46 | a | 2.76 ± 0.61 | a | 2.52 ± 0.8 | b | 3.04 ± 0.19 | a |
| *tetS* | 5.70 | **0.0053** | 2.55 ± 0.71 | a | 2.4 ± 0.77 | a | 1.76 ± 0.94 | b | 2.74 ± 0.45 | a |
| *tetW* | 2.61 | 0.0939 | 2.95 ± 0.49 |  | 2.8 ± 0.6 |  | 2.65 ± 0.72 |  | 2.65 ± 0.72 |  |
| *tetX* | 1.34 | 0.3193 | 1.68 ± 0.95 |  | 1.36 ± 0.91 |  | 2.17 ± 0.82 |  | 1.83 ± 0.88 |  |
| *vanA* | 11.12 | **6E-05** | 0.21 ± 0.35 | b | 0.14 ± 0.35 | c | 0.15 ± 0.37 | b | 0.57 ± 0.64 | a |
| *vanB* | 3.76 | **0.0327** | 1.83 ± 0.63 | ab | 1.76 ± 0.71 | b | 1.75 ± 0.74 | b | 2.17 ± 0.6 | a |

**Supplementary Table 4.** Aligned rank ANOVA with FDR-adjusted p-values and average gene abundances for each soil type.

|  | **Aligned rank ANOVA** | | **Average ± se log copies/g soil** | |
| --- | --- | --- | --- | --- |
| **Gene** | **F.value** | **Adjusted P-value** | **SL** | **SCL** |
| *aacA* | 197 | **1.04E-14** | 2.55 ± 0.75 | 0.25 ± 0.48 |
| *aadA5* | 13.9 | **0.001106** | 2.33 ± 0.73 | 2.56 ± 0.14 |
| *aadD* | 1.16 | 0.329007 | 2.67 ± 0.66 | 2.5 ± 0.19 |
| *acrD* | 59.1 | **5.54E-09** | 2.58 ± 0.64 | 3.14 ± 0.11 |
| *ampC* | 55.8 | **8.34E-09** | 2.41 ± 0.73 | 3.13 ± 0.1 |
| *arr2* | 4.96 | **0.039974** | 0.42 ± 0.51 | 0.58 ± 0.53 |
| *bla_NDM1_* | 6.76 | 0.099107 | 0.61 ± 0.61 | 1.12 ± 0.68 |
| *bla_CTX-M-32_* | 13.5 | **0.018117** | 1.47 ± 0.82 | 0.71 ± 0.68 |
| *bla_imp13_* | 0.39 | **0.001209** | 1.56 ± 0.9 | 0.68 ± 0.62 |
| *bla_KPC_* | 3.15 | 0.569262 | 1.24 ± 0.8 | 1.35 ± 0.76 |
| *bla_NPS_* | 97.4 | **5.64E-12** | 1.47 ± 0.89 | 0.17 ± 0.4 |
| *bla_OXA_* | 0.22 | 0.653288 | 1.65 ± 0.89 | 1.4 ± 0.69 |
| *bla_SHV_* | 4.77 | **0.043042** | 3.14 ± 0.11 | 3.17 ± 0.06 |
| *bla_VIM_* | 7.21 | **0.015513** | 0.22 ± 0.36 | 0.25 ± 0.38 |
| *cadA* | 20.7 | **9.74E-05** | 2.76 ± 0.41 | 2.86 ± 0.07 |
| *catB8* | 8.59 | **0.008368** | 1.26 ± 0.77 | 0.49 ± 0.55 |
| *chrA* | 45.9 | **7.71E-08** | 2.12 ± 0.69 | 2.82 ± 0.32 |
| *cmlB* | 14.9 | **0.000763** | 1.04 ± 0.71 | 1.84 ± 0.55 |
| *copA* | 5.54 | **0.031389** | 1.44 ± 0.82 | 1.98 ± 0.68 |
| *dfr13* | 36.4 | **8.03E-07** | 1.47 ± 0.8 | 0.21 ± 0.35 |
| *ereB* | 113 | **7.21E-13** | 1.36 ± 0.87 | 0.17 ± 0.4 |
| *ermB* | 0 | 0.977171 | 2.34 ± 0.81 | 2.25 ± 0.78 |
| *ermF* | 9.64 | **0.005348** | 2.46 ± 0.73 | 1.44 ± 0.85 |
| *floR* | 4.99 | **0.039974** | 2.62 ± 0.7 | 2.24 ± 0.52 |
| *intI1* | 42.3 | **1.81E-07** | 3.26 ± 0.34 | 2.95 ± 0.31 |
| *IntI2* | 13.3 | **0.001258** | 2.5 ± 0.8 | 1.38 ± 0.78 |
| *IntI3* | 0.37 | 0.569262 | 2.01 ± 0.76 | 1.86 ± 0.68 |
| *mefE* | 6.88 | **0.017678** | 2.62 ± 0.71 | 2.17 ± 0.67 |
| *merA* | 3.3 | 0.093458 | 2.89 ± 0.53 | 2.97 ± 0.31 |
| *mexB* | 0.64 | 0.466056 | 1.76 ± 0.85 | 1.52 ± 0.85 |
| *nikA* | 29.8 | **5.17E-06** | 2.57 ± 0.65 | 3.13 ± 0.12 |
| *qacF* | 20.2 | **0.00011** | 1.71 ± 0.83 | 0.58 ± 0.58 |
| *qnrA* | 2.34 | 0.15553 | 0.57 ± 0.6 | 0.74 ± 0.59 |
| *qnrB* | 11.1 | **0.003006** | 0.75 ± 0.67 | 1.6 ± 0.77 |
| *rcnA* | 58.8 | **5.54E-09** | 2.41 ± 0.71 | 3.06 ± 0.15 |
| *strB* | 38.2 | **5.13E-07** | 3.18 ± 0.33 | 2.32 ± 0.55 |
| *sul1* | 56.4 | **8.19E-09** | 3.33 ± 0.05 | 2.67 ± 0.4 |
| *sul2* | 47.7 | **5.33E-08** | 3.04 ± 0.45 | 1.8 ± 0.81 |
| *sul3* | 17.7 | **0.00026** | 2.42 ± 0.39 | 2.62 ± 0.07 |
| *tetA* | 96.1 | **5.64E-12** | 3.25 ± 0.07 | 3.04 ± 0.07 |
| *tetL* | 0.82 | 0.414417 | 2.69 ± 0.73 | 2.59 ± 0.55 |
| *tetM* | 11.8 | **0.002306** | 2.71 ± 0.67 | 2.94 ± 0.44 |
| *tetS* | 9.91 | **0.004922** | 2.62 ± 0.7 | 2.11 ± 0.78 |
| *tetW* | 6.01 | **0.025533** | 2.99 ± 0.55 | 2.53 ± 0.69 |
| *tetX* | 31.9 | **2.93E-06** | 2.68 ± 0.66 | 0.86 ± 0.77 |
| *vanA* | 28.3 | **8.02E-06** | 0.37 ± 0.54 | 0.17 ± 0.34 |
| *vanB* | 26.8 | **1.24E-05** | 1.38 ± 0.71 | 2.37 ± 0.49 |

**Supplementary Table 5.** 3-way PERMANOVA for 16S microbial community structures

|  | **Df** | **Sums Of Sqs** | **Mean Sqs** | **F Model** | **R^2^** | **Pr(>F)** |
| --- | --- | --- | --- | --- | --- | --- |
| Treatment | 3 | 0.7064 | 0.23545 | 7.58 | 0.07033 | 0.001 |
| Moisture | 2 | 1.0493 | 0.52464 | 16.891 | 0.10447 | 0.001 |
| Type | 1 | 2.3621 | 2.36208 | 76.049 | 0.23518 | 0.001 |
| Treatment:Moisture | 6 | 0.5994 | 0.09989 | 3.216 | 0.05968 | 0.001 |
| Treatment:Type | 3 | 0.5373 | 0.17911 | 5.767 | 0.0535 | 0.001 |
| Moisture:Type | 2 | 1.3055 | 0.65274 | 21.015 | 0.12998 | 0.001 |
| Treatment:Moisture:Type | 6 | 0.6264 | 0.1044 | 3.361 | 0.06237 | 0.001 |
| Residuals | 92 | 2.8575 | 0.03106 | 0.28451 |  |  |
| Total | 115 | 10.0438 | 1 |  |  |  |

**Supplementary Table 6.** Bacterial indicator genera for manure treatments. Groups indicates the manure treatments associated with the genera. Treatments were no manure (NM), antibiotic-free manure (CO), or manure from cattle administered either cephapirin (CE) or pirlimycin (PI). Stat refers to the association of the genus, with higher values corresponding to stronger associations. P-values are FDR adjusted for multiple comparisons.

| **Groups** | **Phylum** | **Class** | **Order** | **Family** | **Genus** | **Stat** | **P-value** |
| --- | --- | --- | --- | --- | --- | --- | --- |
| CE | Firmicutes | Bacilli | Bacillales | Planococcaceae | *Rummeliibacillus* | 0.491 | 0.00887 |
|  | Proteobacteria | Gammaproteobacteria | Xanthomonadales | Xanthomonadaceae | *Luteibacter* | 0.467 | 0.00537 |
|  | Proteobacteria | Betaproteobacteria | Neisseriales | Neisseriaceae | *Formivibrio* | 0.415 | 0.00887 |
|  | Proteobacteria | Alphaproteobacteria | Rhizobiales | Phyllobacteriaceae | *Phyllobacterium* | 0.397 | 0.01432 |
| CO | Actinobacteria | Actinobacteria | Actinomycetales | Corynebacteriaceae | *Corynebacterium* | 0.726 | 0.00537 |
|  | Proteobacteria | Betaproteobacteria | Burkholderiales | Comamonadaceae | *Hydrogenophaga* | 0.497 | 0.02436 |
|  | Euryarchaeota | Methanobacteria | Methanobacteriales | Methanobacteriaceae | *Methanosphaera* | 0.487 | 0.00537 |
|  | Bacteroidetes | Bacteroidia | Bacteroidales | Porphyromonadaceae | *Paludibacter* | 0.483 | 0.00887 |
|  | Chloroflexi | Anaerolineae | Anaerolineales | Anaerolinaceae | *T78* | 0.447 | 0.00887 |
|  | Proteobacteria | Alphaproteobacteria | Rhizobiales | Phyllobacteriaceae | *Aminobacter* | 0.437 | 0.01432 |
|  | Firmicutes | Clostridia | Clostridiales | Peptococcaceae | *Sporotomaculum* | 0.408 | 0.02164 |
|  | Firmicutes | Clostridia | Clostridiales | [Mogibacteriaceae] | *Mogibacterium* | 0.406 | 0.02833 |
|  | Proteobacteria | Alphaproteobacteria | Rhodobacterales | Rhodobacteraceae | *Paracoccus* | 0.365 | 0.04026 |
| NM | Bacteroidetes | Cytophagia | Cytophagales | Cytophagaceae | *Sporocytophaga* | 0.778 | 0.00537 |
|  | Nitrospirae | Nitrospira | Nitrospirales | FW | *29-Apr* | 0.694 | 0.00537 |
|  | Bacteroidetes | [Saprospirae] | [Saprospirales] | Chitinophagaceae | *Segetibacter* | 0.621 | 0.00537 |
|  | Proteobacteria | Betaproteobacteria | Burkholderiales | Comamonadaceae | *Variovorax* | 0.6 | 0.04845 |
|  | Proteobacteria | Alphaproteobacteria | Rhizobiales | Methylobacteriaceae | *Methylobacterium* | 0.597 | 0.01672 |
|  | Proteobacteria | Deltaproteobacteria | Syntrophobacterales | Syntrophaceae | *Smithella* | 0.595 | 0.00537 |
|  | Bacteroidetes | Cytophagia | Cytophagales | Cytophagaceae | *Hymenobacter* | 0.57 | 0.00537 |
|  | Proteobacteria | Alphaproteobacteria | Rhizobiales | Hyphomicrobiaceae | *Parvibaculum* | 0.563 | 0.00887 |
|  | Cyanobacteria | Oscillatoriophycideae | Oscillatoriales | Phormidiaceae | *Phormidium* | 0.535 | 0.00537 |
|  | Firmicutes | Bacilli | Bacillales | Paenibacillaceae | *Paenibacillus* | 0.531 | 0.00537 |
|  | Firmicutes | Clostridia | Clostridiales | Peptococcaceae | *Desulfosporosinus* | 0.525 | 0.00887 |
|  | Firmicutes | Bacilli | Bacillales | Paenibacillaceae | *Ammoniphilus* | 0.522 | 0.00887 |
|  | Cyanobacteria | Nostocophycideae | Nostocales | Nostocaceae | *Nostoc* | 0.499 | 0.00537 |
|  | Proteobacteria | Alphaproteobacteria | Rhodospirillales | Rhodospirillaceae | *Telmatospirillum* | 0.49 | 0.01432 |
|  | Firmicutes | Clostridia | Clostridiales | Peptococcaceae | *Desulfurispora* | 0.475 | 0.02164 |
|  | Firmicutes | Clostridia | Clostridiales | Peptococcaceae | *Pelotomaculum* | 0.471 | 0.00887 |
|  | Cyanobacteria | Synechococcophycideae | Pseudanabaenales | Pseudanabaenaceae | *Leptolyngbya* | 0.454 | 0.01432 |
|  | Firmicutes | Bacilli | Bacillales | Paenibacillaceae | *Brevibacillus* | 0.424 | 0.01200 |
| PI | Firmicutes | Bacilli | Bacillales | Planococcaceae | *Planomicrobium* | 0.99 | 0.00537 |
|  | Proteobacteria | Gammaproteobacteria | Pseudomonadales | Moraxellaceae | *Psychrobacter* | 0.943 | 0.00537 |
|  | Actinobacteria | Actinobacteria | Actinomycetales | Nocardiaceae | *Rhodococcus* | 0.79 | 0.00537 |
|  | Firmicutes | Clostridia | Clostridiales | Peptostreptococcaceae | *Clostridium* | 0.58 | 0.00537 |
|  | Firmicutes | Bacilli | Lactobacillales | Carnobacteriaceae | *Desemzia* | 0.552 | 0.00537 |
|  | Proteobacteria | Gammaproteobacteria | Xanthomonadales | Xanthomonadaceae | *Stenotrophomonas* | 0.537 | 0.00537 |
|  | Spirochaetes | Spirochaetes | Spirochaetales | Spirochaetaceae | *Treponema* | 0.371 | 0.02164 |
| CE+CO | Firmicutes | Clostridia | Clostridiales | Ruminococcaceae | *Ruminococcus* | 0.507 | 0.01200 |
|  | Tenericutes | Mollicutes | Anaeroplasmatales | Anaeroplasmataceae | *Asteroleplasma* | 0.504 | 0.00537 |
|  | Actinobacteria | Actinobacteria | Actinomycetales | Promicromonosporaceae | *Cellulosimicrobium* | 0.391 | 0.04648 |
| CE+NM | Proteobacteria | Deltaproteobacteria | Myxococcales | Polyangiaceae | *Aetherobacter* | 0.476 | 0.04650 |
| CE+PI | Euryarchaeota | Thermoplasmata | E2 | [Methanomassiliicoccaceae] | *Methanomassiliicoccus* | 0.512 | 0.01200 |
| CO+PI | Firmicutes | Bacilli | Turicibacterales | Turicibacteraceae | *Turicibacter* | 0.985 | 0.00537 |
|  | Proteobacteria | Alphaproteobacteria | Caulobacterales | Caulobacteraceae | *Mycoplana* | 0.713 | 0.00537 |
|  | Proteobacteria | Betaproteobacteria | Burkholderiales | Comamonadaceae | *Rubrivivax* | 0.702 | 0.00537 |
|  | Firmicutes | Clostridia | Clostridiales | Lachnospiraceae | *Epulopiscium* | 0.638 | 0.00537 |
|  | Proteobacteria | Deltaproteobacteria | Myxococcales | Polyangiaceae | *Chondromyces* | 0.498 | 0.01672 |
|  | Actinobacteria | Actinobacteria | Actinomycetales | Dietziaceae | *Dietzia* | 0.432 | 0.02164 |
|  | Firmicutes | Bacilli | Bacillales | Staphylococcaceae | *Jeotgalicoccus* | 0.412 | 0.01974 |
|  | Firmicutes | Bacilli | Lactobacillales | Aerococcaceae | *Facklamia* | 0.412 | 0.03074 |
| CE+CO  +NM | Proteobacteria | Alphaproteobacteria | Rhodospirillales | Rhodospirillaceae | *Skermanella* | 0.706 | 0.01430 |
| CE+CO  +PI | Proteobacteria | Gammaproteobacteria | Pseudomonadales | Moraxellaceae | *Acinetobacter* | 0.959 | 0.00537 |
|  | Firmicutes | Clostridia | Clostridiales | Clostridiaceae | *SMB53* | 0.959 | 0.00537 |
|  | Proteobacteria | Deltaproteobacteria | Myxococcales | Polyangiaceae | *Sorangium* | 0.881 | 0.00537 |
|  | Bacteroidetes | Flavobacteriia | Flavobacteriales | Flavobacteriaceae | *Flavobacterium* | 0.858 | 0.00537 |
|  | Proteobacteria | Alphaproteobacteria | Sphingomonadales | Sphingomonadaceae | *Sphingobium* | 0.854 | 0.00537 |
|  | Gemmatimonadetes | Gemmatimonadetes | Gemmatimonadales | Gemmatimonadaceae | *Gemmatimonas* | 0.83 | 0.00537 |
|  | Euryarchaeota | Methanobacteria | Methanobacteriales | Methanobacteriaceae | *Methanobrevibacter* | 0.822 | 0.00537 |
|  | Bacteroidetes | Sphingobacteriia | Sphingobacteriales | Sphingobacteriaceae | *Pedobacter* | 0.813 | 0.00537 |
|  | Bacteroidetes | Cytophagia | Cytophagales | Cytophagaceae | *Dyadobacter* | 0.805 | 0.00537 |
|  | Proteobacteria | Alphaproteobacteria | Caulobacterales | Caulobacteraceae | *Caulobacter* | 0.767 | 0.00537 |
|  | Bacteroidetes | [Saprospirae] | [Saprospirales] | Chitinophagaceae | *Chitinophaga* | 0.759 | 0.02623 |
|  | Proteobacteria | Deltaproteobacteria | Myxococcales | Nannocystaceae | *Nannocystis* | 0.73 | 0.00537 |
|  | Proteobacteria | Betaproteobacteria | Methylophilales | Methylophilaceae | *Methylotenera* | 0.676 | 0.03859 |
|  | Firmicutes | Clostridia | Clostridiales | Lachnospiraceae | *Coprococcus* | 0.662 | 0.02623 |
|  | Proteobacteria | Alphaproteobacteria | Rhodobacterales | Rhodobacteraceae | *Rhodobacter* | 0.651 | 0.00537 |
|  | Proteobacteria | Alphaproteobacteria | Sphingomonadales | Sphingomonadaceae | *Sphingopyxis* | 0.648 | 0.00537 |
|  | Bacteroidetes | [Saprospirae] | [Saprospirales] | Chitinophagaceae | *Flavihumibacter* | 0.622 | 0.00537 |
|  | Bacteroidetes | [Saprospirae] | [Saprospirales] | Chitinophagaceae | *Lacibacter* | 0.603 | 0.00537 |
|  | Firmicutes | Clostridia | Clostridiales | Ruminococcaceae | *Clostridium* | 0.602 | 0.01672 |
|  | Bacteroidetes | Flavobacteriia | Flavobacteriales | [Weeksellaceae] | *Chryseobacterium* | 0.594 | 0.01200 |
|  | Bacteroidetes | Flavobacteriia | Flavobacteriales | Cryomorphaceae | *Crocinitomix* | 0.584 | 0.00537 |
|  | Firmicutes | Clostridia | Clostridiales | Veillonellaceae | *Sporomusa* | 0.58 | 0.04239 |
|  | Euryarchaeota | Methanobacteria | Methanobacteriales | Methanobacteriaceae | *Methanobacterium* | 0.577 | 0.01432 |
|  | Proteobacteria | Gammaproteobacteria | Xanthomonadales | Xanthomonadaceae | *Pseudoxanthomonas* | 0.553 | 0.04026 |
|  | Proteobacteria | Betaproteobacteria | Burkholderiales | Comamonadaceae | *Acidovorax* | 0.544 | 0.01672 |
|  | Verrucomicrobia | Verrucomicrobiae | Verrucomicrobiales | Verrucomicrobiaceae | *Luteolibacter* | 0.544 | 0.01200 |
|  | Firmicutes | Clostridia | Clostridiales | [Tissierellaceae] | *Sedimentibacter* | 0.533 | 0.02833 |
|  | Spirochaetes | Spirochaetes | Spirochaetales | Spirochaetaceae | *Spirochaeta* | 0.532 | 0.02623 |

**Supplementary Table 7.** 3-way ANOVA for respiration

|  | **Df** | **Sum Sq** | **Mean Sq** | **F value** | **Pr(>F)** |
| --- | --- | --- | --- | --- | --- |
| Treatment | 3 | 20.331 | 6.777 | 684.488 | < 2e-16 |
| Moisture | 2 | 2.345 | 1.172 | 118.418 | < 2e-16 |
| Type | 1 | 0.151 | 0.151 | 15.223 | 0.000177 |
| Treatment:Moisture | 6 | 0.986 | 0.164 | 16.604 | 4.78E-13 |
| Treatment:Type | 3 | 0.126 | 0.042 | 4.255 | 0.00723 |
| Moisture:Type | 2 | 6.728 | 3.364 | 339.757 | < 2e-16 |
| Treatment:Moisture:Type | 6 | 2.534 | 0.422 | 42.651 | < 2e-16 |
| Residuals | 96 | 0.95 | 0.01 |  |  |

**Supplementary Table 8.** 3-way ANOVA for Substrate Induced Respiration (SIR)

|  |  | **Df** | **Sum Sq** | **Mean Sq** | **F value** | **Pr(>F)** |
| --- | --- | --- | --- | --- | --- | --- |
| Treatment |  | 3 | 33.09 | 11.03 | 98.849 | < 2e-16 |
| Moisture |  | 2 | 5.35 | 2.68 | 23.975 | 3.59E-09 |
| Type |  | 1 | 33.84 | 33.84 | 303.276 | < 2e-16 |
| Treatment:Moisture |  | 6 | 3.64 | 0.61 | 5.431 | 7.17E-05 |
| Treatment:Type |  | 3 | 5.86 | 1.95 | 17.505 | 3.82E-09 |
| Moisture:Type |  | 2 | 27.39 | 13.7 | 122.743 | < 2e-16 |
| Treatment:Moisture:Type |  | 6 | 8.54 | 1.42 | 12.76 | 1.52E-10 |
| Residuals |  | 96 | 10.71 | 0.11 |  |  |

**Supplementary Table 9.** Average (n=3) ± SD NO_3_ and NH_4_ in soil and manure that was added to the mesocosms

|  | NO_3_ ppm | NH_4_ ppm |
| --- | --- | --- |
| Sand | 3.96 ± 0.14 | 3.44 ± 0.28 |
| Clay | 1.64 ± 0.04 | 8.05 ± 0.39 |
| CO | 24.4 ± 10.35 | 611.89 ± 89.42 |
| CE | 29.47 ± 0.56 | 258.36 ± 27.28 |
| PI | 29.52 ± 2.28 | 421.65 ± 19.77 |


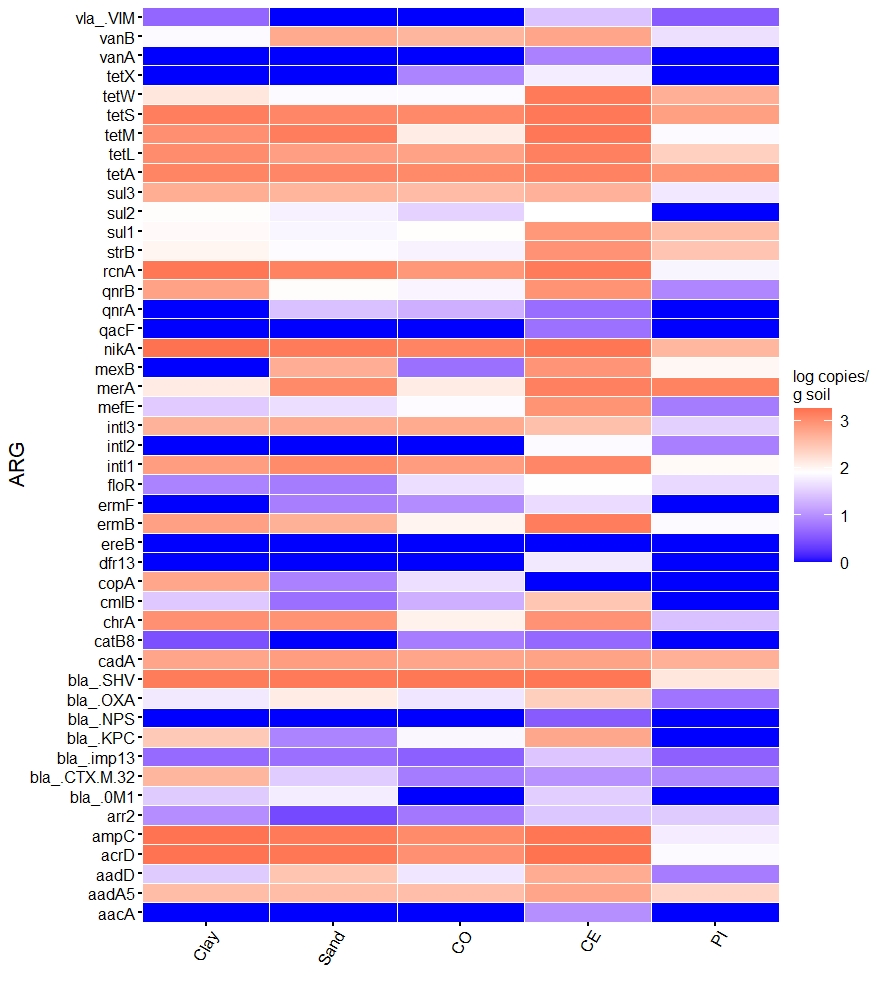


**Supplementary Figure 1.** Background average abundance (log copies/g) of ARGs measured via mfqPCR in soil and manure after 10 weeks in a sandy clay loam (clay) soil, sandy loam (sand) soil, antibiotic-free manure (CO), or manure from cattle administered either cephapirin (CE) or pirlimycin (PI).


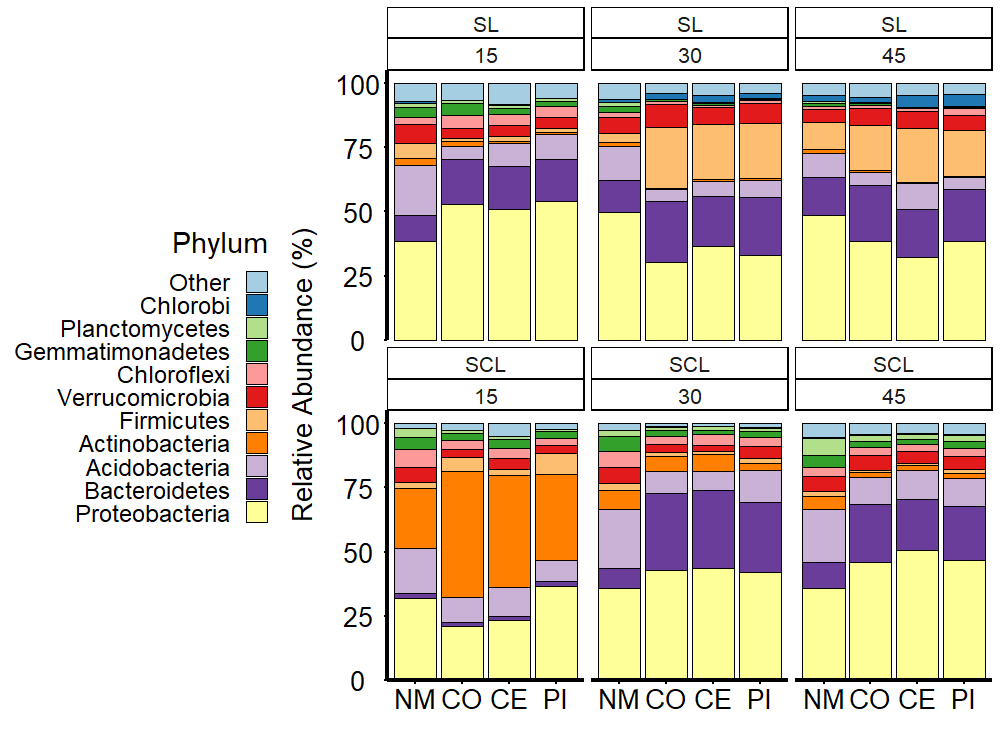
 **Supplementary Figure 2.** Average relative abundance (%) of the 10 most abundant phyla in soil bacterial communities after 10 weeks in a sandy clay loam (SCL) or sandy loam (SL) soil. Soils were kept at 15, 30, or 45% moisture content, treatments of no manure (NM), antibiotic-free manure (CO), or manure from cattle administered either cephapirin (CE) or pirlimycin (PI) in was added every other week.


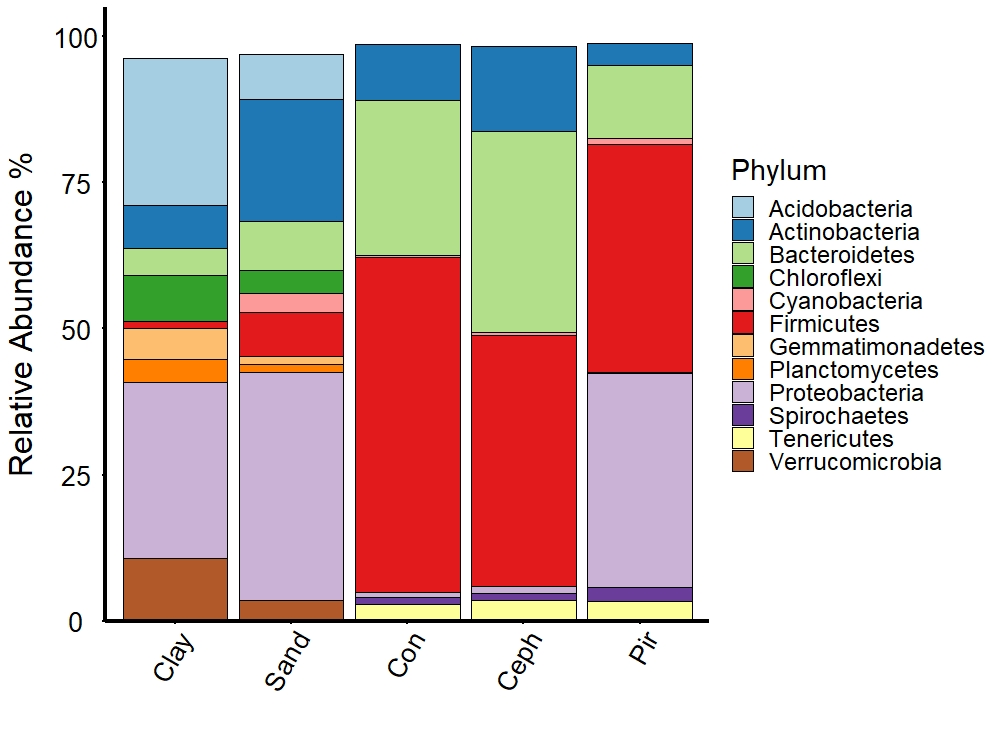


**Supplementary Figure 3.** Mean relative abundance (%) of the 12 most abundant phyla in bacterial communities in sandy clay loam (SCL) or sandy loam (SL) soil and in the manure from antibiotic-free cattle (Con), cattle administered cephapirin (Ceph), or cattle administered pirlimycin (Pir) before the start of the experiment.


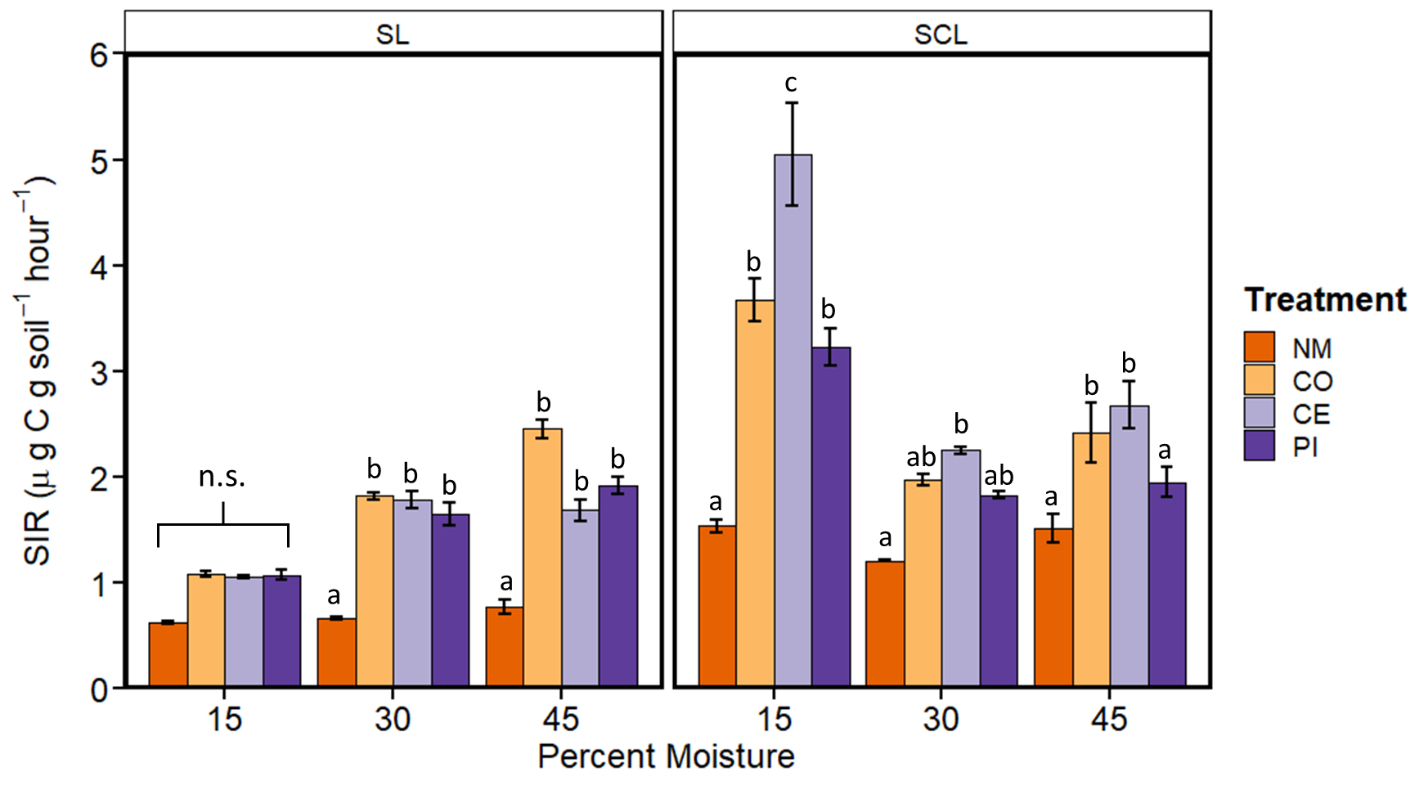


**Supplementary Figure 4.** Average ± SE (n=5) SIR in a sandy clay loam (SCL) or sandy loam (SL) soil kept at 15, 30, or 45% moisture content by mass, and treatments of no manure (NM), antibiotic-free manure (CO), or manure from cattle administered either cephapirin (CE) or pirlimycin (PI) was added every other week for 10 weeks prior to SIR analysis. Lowercase letters indicate statistical significance of treatments within moisture and soil type groups. N.s. = no statistical significance.


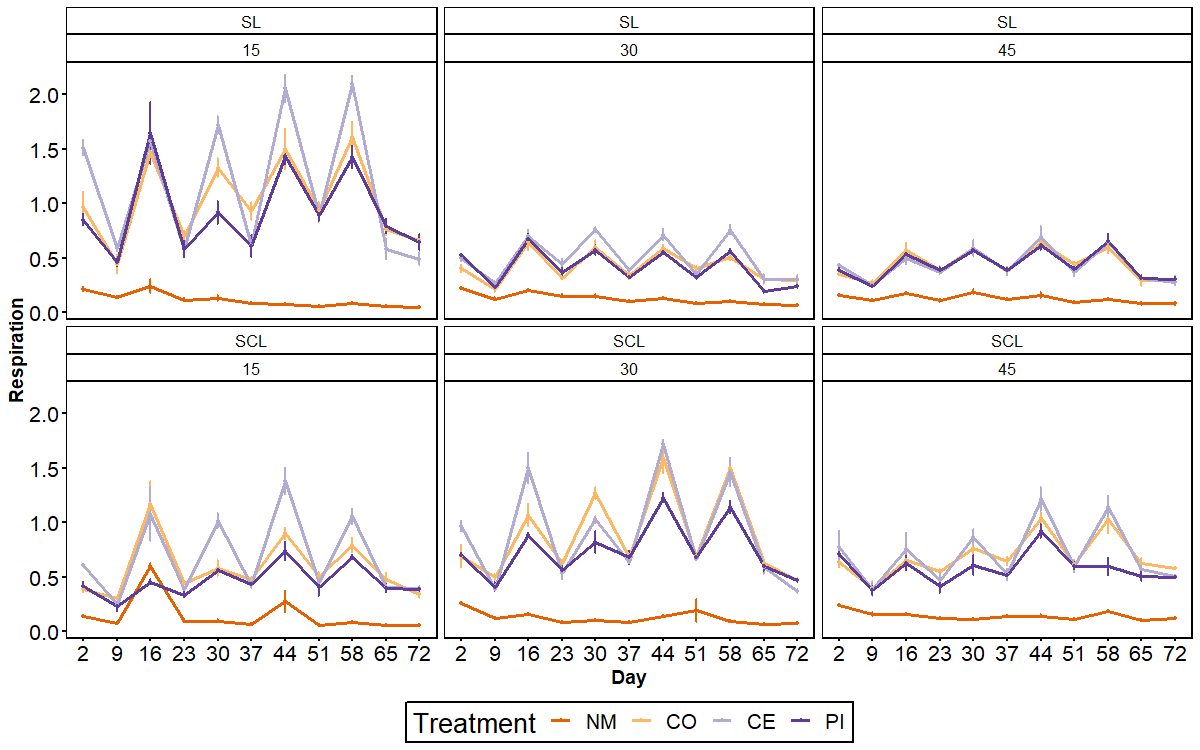


**Supplementary Figure 5.** Average ± SE (n=5) respiration over 10 weeks in a sandy clay loam (SCL) or sandy loam (SL) soil. Soils were kept at 15, 30, or 45% moisture content by mass, and treatments of no manure (NM), antibiotic-free manure (CO), or manure from cattle administered either cephapirin (CE) or pirlimycin (PI) was added every other week.
